# Smart Mixture Design Can Steer the Fate of Root‐Derived Carbon Into Mineral‐Associated and Particulate Organic Matter in Intensively Managed Grasslands

**DOI:** 10.1111/gcb.70117

**Published:** 2025-03-06

**Authors:** Esben Øster Mortensen, Diego Abalos, Tine Engedal, August Kau Lægsgaard, Kirsten Enggrob, Carsten W. Mueller, Jim Rasmussen

**Affiliations:** ^1^ Department of Agroecology Aarhus University Tjele Denmark; ^2^ CBIO Aarhus University Centre for Circular Bioeconomy Aarhus University Tjele Denmark; ^3^ iCLIMATE Interdisciplinary Centre for Climate Change Aarhus University Roskilde Denmark; ^4^ Department of Plant and Environmental Sciences University of Copenhagen Frederiksberg Denmark; ^5^ Institute of Ecology, Chair of Soil Science Technische Universität Berlin Berlin Germany; ^6^ Department of Geosciences and Natural Resource Managements University of Copenhagen Copenhagen Denmark

**Keywords:** ^13^C isotopic labelling, forbs, Grasses, legumes, plant functional groups, root traits, soil carbon fractionation, soil carbon storage

## Abstract

Species choice and richness in intensively managed grassland mixtures regulate soil carbon (C) input via rhizodeposition, with potential consequences for long‐term soil organic carbon storage. Based on a field trial with different grass–legume–forb mixtures, we removed roots from the soil, which was then subjected to particle‐size fractionation to trace fresh organic carbon (net C rhizodeposition) into particulate organic matter (POM) and mineral‐associated organic matter (MAOM). We related these C input fractions to root traits. Using multiple‐pulse ^13^C–CO_2_‐labeling, we captured the net formation of mineral‐associated organic carbon (MAOC) and particulate organic carbon (POC) at the end of the growing season. Pure stand perennial ryegrass ( *Lolium perenne* ) had higher quantities of rhizodeposited C allocated to MAOC and POC (0.21 and 0.13 g C kg^−1^ dry soil, respectively) compared to grass–legume–forb mixtures (ranging from 0.10 to 0.12 for MAOC and 0.05 to 0.06 g C kg^−1^ dry soil for POC). However, the proportion of MAOC (%MAOC of net C rhizodeposition) in relation to that of POC was higher in mixtures with legumes. Species richness did not affect the quantity of MAOC or POC, nor %MAOC. The quantities of MAOC and POC were positively associated with root length. In contrast, %MAOC was positively associated with root diameter and a lower root C:N ratio. Despite higher %MAOC in mixtures with legumes, the main driver of MAOC and POC quantities was the total amount of C rhizodeposition. These results highlight the importance of legumes in the formation of MAOC from rhizodeposition and of high root length for increasing both MAOC and POC quantities. Our study shows how plant community design can be used to increase MAOC and/or POC and facilitate soil C storage. By revealing the traits behind the relationships between plant communities and MAOC and POC formation, we provide a guide for species selection in intensively managed grasslands to mitigate climate change.

## Graphical abstract

Grassland species and specific root traits affect the allocation of root‐derived carbon into soil organic matter pools with varying persistence, i.e., mineral‐associated organic matter (MAOC) and particulate organic matter (POC) in intensively managed grasslands. We found that legumes increased the proportion of MAOC, while grasses increased the proportion of POC and total net rhizodeposition. By identifying root traits that can steer the formation of specific soil C pools, this study presents a biological climate mitigation tool that can be used globally in managed grasslands and beyond the species and mixtures tested in our study.

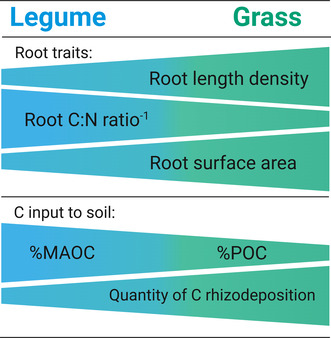

## 1 Introduction

Increasing and maintaining rather persistent soil organic carbon (SOC) in agricultural soils are crucial for soil fertility and climate change mitigation (Lal 2004, 2010; Sanderman et al. 2017; Smith et al. 2015, 2020). Perennial grasslands can play an important role in efforts to store more organic carbon (OC), primarily due to high root carbon inputs from grassland species with elevated SOC stabilization efficiency (Bai and Cotrufo 2022; Jensen et al. 2022; Ledo et al. 2020). Within grassland systems, increasing the number of plant species promotes aboveground productivity at lower fertilizer inputs and may further stimulate SOC storage compared to monocultures, particularly when legumes are present (Chen et al. 2018, 2020). However, we still have an incomplete understanding of the mechanisms by which grassland species mixtures increase SOC and of the main plant features driving such effects.

Particulate organic matter (POM) and mineral‐associated organic matter (MAOM) pools are widely different in terms of their bioavailability and thus persistence, with MAOM contributing more to longer‐term soil carbon storage (Cotrufo et al. 2019; Lavallee et al. 2020; Lehmann and Kleber 2015). Carbon rhizodeposition is a key source of MAOM after microbial transformations (Liang et al. 2019) or directly through the adsorption of soluble rhizodeposits to minerals without microbial transformation (Mikutta et al. 2019; Sokol et al. 2018; Teixeira et al. 2024), whereas around 20% of root litter turns into POM (Villarino et al. 2021). Accordingly, plant species differing in rhizodeposition rates can have different impacts on MAOM and POM (Henneron et al. 2019). Species that contribute more to POM buildup may induce higher soil aggregation, which in turn enhances the physical protection of SOC (Freschet et al. 2021; Poirier et al. 2018). Since species mixtures are associated with higher root biomass and exudation compared to monocultures (Chen et al. 2020), they may increase SOC stabilization by stimulating the formation of MAOM, e.g., due to more diverse C rhizodeposits (Eisenhauer et al. 2017; Lange et al. 2019). Increased rhizodeposition may also promote a priming effect by stimulating microbial turnover of existing SOM (Huo et al. 2017), but the positive effect on at least MAOM formation is likely to be higher due to the easily accessible C and N in rhizodeposits (Villarino et al. 2021), although the overall effect on the C balance depends on the specific soil C status (Angst et al. 2023). Earlier experiments have investigated the effect of species richness on soil dynamics such as nutrient cycling, root C input to soil, microbial growth, and microbial carbon use efficiency in semi‐natural ecosystems without fertilization (e.g., Furey and Tilman 2021; Lange et al. 2015; Prommer et al. 2020). However, the fate of C rhizodeposition and SOC stabilization remains understudied in intensively managed grasslands, which are temporary (< 5 years), harvested systems managed for maximum productivity with irrigation and mineral fertilization, often based on pure stands of nutrient‐acquisitive species.

Plants can be categorized according to their traits into functional groups such as grasses, forbs, and legumes (Barneze et al. 2024; Fernandez Pulido et al. 2023). Recent studies have shown that root morphological and biochemical traits can be strong predictors of rhizodeposition and of soil C processes (Bardgett et al. 2014; Engedal et al. 2023; Henneron et al. 2019). Grasses are characterized by high specific root length (SRL) and low average root diameter (ARD) (Gould et al. 2016). These fine roots have lower turnover time, resulting in a higher input of organic matter (OM) to the soil (Goebel et al. 2011; Gould et al. 2016; Liang et al. 2016), which could lead to both increased MAOM and POM. Legumes and some forbs have a higher root diameter and lower SRL, traits that facilitate mycorrhizal colonization (Sweeney et al. 2021). This, in turn, can increase net rhizodeposition of the colonized root (Zhou et al. 2020) and favor the formation of MAOM through microbial (in vivo) turnover (Liang et al. 2017). Legumes have lower C:N ratios than grasses and forbs due to the capability of legumes to fix N_2_ from the atmosphere. These lower C:N ratios increase the quality of rhizodeposition, which may favor MAOM buildup due to stimulation of microbial activity (Peixoto et al. 2022). Legume biomass also has a higher proportion of lignin compared to grasses and forbs (Hoffman et al. 1993; Solati et al. 2017), and higher lignin content may result in higher formation of POM compared to MAOM due to slower decomposition. Differences in lignin content can also be present within legume species, with white clover ( *Trifolium repens* ) having lower lignin content than red clover ( *Trifolium pratense* ) in root biomass (Louarn et al. 2014). Collectively, these results suggest that it may be possible to combine plants with different functional traits to maximize C inputs to both mineral‐associated OC (MAOC) and particulate OC (POC). Revealing the link between root traits and the allocation of newly formed SOC into MAOC and POC pools can shed light on this knowledge gap.

Here, we investigated the fate of ^13^C from rhizodeposition into SOC fractions (i.e., MAOC and POC) in a range of intensively managed grassland mixtures and related this to specific root traits. We selected five treatments (Table 1) representing increasing plant species diversity: (i and ii) pure stand perennial ryegrass ( *Lolium perenne* ) at low and high N fertilization, (iii) a two‐species mixture with perennial ryegrass and white clover representing a common productive mixture with well‐documented mutualistic interactions between the two species (e.g., Rasmussen et al. 2013), (iv) a multi‐species mixture (grass–legume–forb) with six species having strong agronomic performance (Cong et al. 2017; Cougnon et al. 2014; Dhamala et al. 2016), and (v) a multi‐species mixture with 18 species simulating the number of species shown to generate positive mixture effects in natural grasslands (Isbell et al. 2011; Lange et al. 2023). The accumulated net rhizodeposited C was measured at the end of the main growing season. We consider rhizodeposition to be compounds released from the root, i.e., exudates, mucilage, sloughed off root cells, and turnover of finer root parts of the living plant (Jones et al. 2004; Rasmussen 2011). Thus, any root fragments detached from the living plant due to mechanical disturbance during sampling are excluded from estimation of rhizodeposition (Mortensen et al. 2021). In the present study, C rhizodeposition and allocation to MAOC and POC fractions were measured during the living‐plant phase of a temporal grassland; consequently, the longer‐term fate of the standing root biomass was not considered.

**TABLE 1:** Treatment overview with N fertilizer rate and sown species. All species included in Mix18, as well as seeding density and fertilization scheme for all treatments can be found in Table S1.

Treatment	Fertilizer (kg N ha^−1^ year^−1^)	Species
*Grass300N*	*300*	*Perennial ryegrass*
Grass75N	75	Perennial ryegrass
Mix2	75	Perennial ryegrass, white clover
Mix6	75	Perennial ryegrass, tall fescue, white clover, red clover, chicory, plantain
Mix18	75	Mix6 + additionally 12 species (in total 6 grasses, 8 legumes, and 4 forbs)

*Note:* Scientific names: perennial ryegrass ( *Lolium perenne* ), tall fescue ( *Festuca arundinacea* ), white clover (*Trifolium repense*), red clover ( *Trifolium pratense* ), and the forbs chicory ( *Cichorium intybus* ) and plantain ( *Plantago lanceolata* ).

The aim of the study was to investigate the fate of rhizodeposited C into SOC fractions with varying expected persistence (i.e., MAOC and POC) as affected by grassland species richness and functional groups (grass, legumes, and forbs), and to investigate the root traits driving these effects.

We hypothesized that:

1. Plant communities with higher SRL (i.e., lower ARD) will increase both the quantity of POC and the relative proportion of rhizodeposition recovered as POC because smaller roots are the key source of POM in living swards.
2. Higher species richness will increase the proportion of rhizodeposition recovered as MAOC compared to POC due to a more diverse plant input leading to an increased microbial turnover rate and efficiency.
3. Inclusion of legumes will increase the proportion of rhizodeposition recovered as MAOC compared to pure stand perennial ryegrass due to more N‐rich root biomass stimulating microbial activity.

## 2 Materials and Methods

### 2.1 Site, Experimental Setup, and Isotopic Labeling to Trace C Input to Soil

A field experiment was initiated in the autumn of 2021 at AU Viborg, Denmark (56°30′ N, 9°34′ E). The field had a history of several years of a conventional grain crop rotation, alternating between winter cereals (rye and wheat) and spring cereals (oat and barley). Soil type was a Mollic Luvisol (according to WRB (2022)) with 364 g coarse sand kg^−1^, 303 g fine sand kg^−1^, 239 g silt kg^−1^, 70 g clay kg^−1^, 16 g organic C kg^−1^, 1.4 g N kg^−1^, a C:N ratio of 11.6, and a pH of 5.24.

Five treatments along a species richness gradient were used (Table 1): two pure ryegrass stands with different fertilization regimes and mixed stands with ryegrass and different numbers of either clover or other plant species, up to 18 species in total. All treatments had four replicates arranged in four blocks in a completely randomized block design. The two treatments with pure stand perennial ryegrass were fertilized with 75 and 300 kg N ha^−1^ year^−1^ (thus referred to as Grass75N and Grass300N). The high‐fertilized treatment represents common practice for intensively managed grasslands in Denmark, while the low‐fertilized treatment had the same N rate as the three mixtures. The mixtures comprised a two‐species mixture with ryegrass and white clover (Mix2) and two multi‐species mixtures with 6 and 18 species (Mix6 and Mix 18) including grasses, legumes, and forbs (Table S1). All three mixtures were fertilized with 75 kg N ha^−1^ year^−1^ to stimulate the growth of non‐legume species. The experiment was managed according to best agricultural practice, i.e., fertilized with an optimal amount of P and K, irrigated during dry summer periods, and with a low‐frequency cutting regime resulting in high biomass yields (three cuts per year).

During 2022, multiple‐pulse ^13^C–CO_2_‐labeling was performed from April 1 (start of the growing period) until mid‐October (last biomass cut) in PVC cylinders (inner diameter 29.5 cm) that were inserted 25 cm into the soil in spring before the growing season (Figure S1). This was done to quantify the net C input to soil lost from the living plant via rhizodeposition. For plant labeling, the fully ^13^C‐labeled sodium carbonate (99 atom%) was dissolved in a 0.1 M sodium hydroxide solution at a concentration of 50 g ^13^C‐Na_2_CO_3_ L^−1^. The ^13^C solution was added to a beaker inside the cylinder, which, during the labeling event, was closed with a transparent plastic bag. Surplus HCl was then added to release ^13^C‐CO_2_ for assimilation via photosynthesis. After half an hour, the unlabeled sodium carbonate in a 0.1 M sodium hydroxide solution (50 g Na_2_CO_3_ L^−1^) was added to the beaker to enhance plant assimilation of ^13^CO_2_ by maintaining a sufficiently high CO_2_ level. The amount of labeling solution was adjusted according to the increasing plant biomass and assumed increase in photosynthesis. The ^13^CO_2_‐enriched atmosphere remained over the cylinder between 1 and 3 h during each labeling session, depending on temperature and sunshine conditions.

In October 2022, cylinders were excavated and separated into shoot, root, and soil pools. Total root biomass to 25‐cm depth was quantified for the entire cylinder core (29.5 cm diameter) in three steps. Roots attached to the remaining aboveground biomass after the last cut (referred to as stubble) were separated carefully from the soil and split from the stubble before they were washed to differentiate the ^13^C in roots from the plant‐derived ^13^C in soils adhering to the roots at sampling. The soil was then sieved (10 mm), and roots left on top of the sieve were cleaned and added to the root fraction. Lastly, the soil was thoroughly mixed, and approx. 1.2 kg was passed through a 2‐mm sieve, and the smaller roots caught on the 2‐mm sieve were collected. From the 2‐mm sieved soil, subsamples were taken for bulk isotope analysis, root fragment determination (Mortensen et al. 2021), and SOC fractionation. The soil subsamples for fractionation were stored at −20°C after excavation. On February 17, 2023, these samples were thawed and immediately sieved through a 2‐mm sieve and dried for 5 days at 40° until stable weight.

### 2.2 Fractionation of Recently Formed C Into Soil Organic Carbon Fractions

A particle‐size fractionation protocol was used to partition photosynthate‐derived ^13^C in the recently formed SOM via wet sieving of the dried soil samples into what was defined as MAOC (< 50 μm), POC (50–250 μm), and root fragments (> 250 μm), after the soil samples had been dispersed with a probe‐tube sonicator (B. Braun, Labsonic U). Although the size separation does not ensure a complete separation of OM particles (e.g., light pure plant and/or microbial residues) and mineral‐associated OM sensu stricto, we follow the naming of POM (50–250 μm) and MAOM (> 50 μm) of these size fractions according to other recent studies (e.g., Engedal et al. 2023; Lavallee et al. 2020; Zhang et al. 2022). For the largest fraction (> 250 μm) we consider that the major part of the SOC consisted of fresh root fragments detached during soil sampling (Mortensen et al. 2021).

In a beaker, 30 g of sieved dry soil was capillary saturated in 150 mL MiliQ water (1:5 soil water ratio) for 15 min, prior to ultrasonication at 250 J/mL (calibration according to Schmidt et al. (1999)). The ultrasonication energy was set to a level that was demonstrated before to disperse sandy soils without leading to a translocation of organic particles into smaller sized fractions (Amelung and Zech 1999; Cerli et al. 2012; Christensen 1992; Engedal et al. 2023; Mueller et al. 2014). The probe was inserted 1.5 cm into the soil suspension, and the beaker was placed in an ice water bath during ultrasonication to ensure that the suspension temperature did not increase above 60°C. After dispersion and wet sieving, all fractions were oven‐dried at 60°C and homogenized via ball milling. For total C, N, and ^13^C determination, 20–30 mg of soil sample was packed in tin capsules and analyzed using a Flash Elemental Analyser (Thermo Scientific, Hvidovre, Denmark) coupled via a thermal conductivity detector (TCD) to an isotope ratio mass spectrometer (Delta V Plus IRMS, Thermo Scientific, Hvidovre, Denmark). There were no differences between treatments in total C or N content in bulk soil, POM, or MAOM fractions (Table S2). Across treatments, the C recoveries after fractionation were 3.9%–4.8%, 13.7%–14.8%, and 71.2%–76.2% for root fragments, POM, and MAOM fractions respectively, and the total % C recovery after fractionation ranged from 90 ± 4 to 96 ± 3 (Table S2).

We compared the quantity of ^13^C in root fragments (> 250 μm) recovered in the present study using the fractionation protocol to the quantity found via decantation and wet sieving with visual inspection (Mortensen, Abalos, Rasmussen 2025). The two protocols had the same cutoff (> 250 μm) and subsamples from the same treatments were used. We found a correlation between the ^13^C quantity in root fragments recovered with the two protocols (*p* = 0.046; *R* = 0.45, Figure S2), although a lower variance among replicates was found in the present study. This supports our distinction that the OC fraction above 250 μm predominantly consisted of fresh root fragments detached during sampling. Thus, the root fragments (> 250 μm) were recognized as part of the standing root biomass at sampling time in accordance with Liang et al. (2022). The newly derived C < 250 μm was recognized as net C rhizodeposition and divided into MAOC (< 50 μm) and POC (> 50 μm), totaling 100% of rhizodeposition (%MAOC + %POC). Analysis of the C:N ratio in MAOC and POC fractions showed qualitative differences, where the MAOC fraction had a significantly lower C:N ratio in all treatments compared to the POC fraction (Figure S3), indicating a higher content of microbially derived SOC in the MAOC fraction in contrast to the assumed more plant‐derived C in the POC fraction (Angst et al. 2023).

### 2.3 Root Trait Analysis

Morphological root traits were obtained by scanning subsamples of cleaned roots before drying (EPSON Perfection V700/V750 3.92), and images were analyzed by the software WINRHIZO (Regent Instruments Inc., Quebec City, QC, Canada). Based on the data output from WINRHIZO and the dry matter (DM) content of roots or soil, the following root traits were calculated: Specific Root Length (SRL, [m root/g DM root]), Root Length Density (RLD, [cm root/cm^3^ of dry soil]), Average Root Diameter (ARD, [mm]), Root Surface Area (RSA, [cm^2^ root/g DM root]), and Root Tissue Density (RTD, [g DM root/cm^3^ root]). All roots were dried, ground, and prepared for further analysis. From the ground samples, biochemical root traits were obtained as the measured root C:N ratio, as well as the lignocellulose index based on the content of lignin, cellulose, and hemicellulose in dry root biomass (Herman et al. 2008) calculated as (*Lignin/(Lignin* + *cellulose* + *hemicellulose*)). The content of lignin, cellulose, and hemicellulose in root biomass was derived by a FOSS Fibertec 2010 digester, using a neutral detergent to derive the total neutral detergent fiber fraction (lignin, cellulose and hemicellulose). An acid detergent was used to separate hemicellulose from cellulose and lignin, and finally, cellulose was separated from lignin using 72% sulfuric acid (Cong et al. 2015). The C:N ratio was based on the C and N content in root biomass obtained by the same method as described above for soil C and N content, using 2–3 mg of ball‐milled, representatively sampled, dried root biomass packed in tin capsules.

### 2.4 Calculation of Net C Rhizodeposition

The pool of net C rhizodeposition was divided into the MAOC and POC fractions. The net C lost via rhizodeposition from the living plant (ClvR) to the soil was quantified by a tracer mass balance approach, following the terminology by Rasmussen et al. (2019), Mortensen et al. (2021) and Engedal et al. (2023). Note that the term “C lost” refers to the plant perspective, due to the tracer mass balance approach that is based on the assimilated C in the plant pools, but from a soil C storage and sequestration perspective, this C cannot be considered necessarily lost. Since C rhizodeposition is part of the total assimilated C in plant biomass, the relative C lost via rhizodeposition (%ClvR) was calculated based on ^13^C quantity in roots and not in the soil pool (Rasmussen et al. 2019). Thus, %ClvR was expressed and calculated as a percentage of the ^13^C recovered in roots and soil:

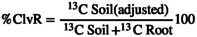

where the ^13^C in soil was adjusted for the ^13^C in root fragments (Mortensen et al. 2021). The ^13^C soil pool was calculated by multiplying the atom% ^13^C excess of soil by the amount of total C in the soil sample, while the ^13^C root pool was calculated by multiplying the atom% ^13^C excess of root by the amount of total C in the root sample. Based on the %ClvR and total quantity root C, the quantity of C lost via rhizodeposition (qClvR) expressed in g C kg^−1^ dry soil was calculated as:

$$\text{qClvR}=\frac{\%\text{ClvR}\times \text{rootC}\;}{100-\%\text{ClvR}}$$

### 2.5 Data Analysis and Statistics

Data were explored and analyzed in R version 4.3.2 (R Core Team 2023) and are available in an online repository (Mortensen, Abalos, Enggrob et al. 2025). Linear mixed‐effects models were fitted for all root traits with the package lme4 (Bates et al. 2015), and where the assumptions were not met, generalized linear mixed‐effects models were fitted using the package glmmTMB (Brooks et al. 2017) with a gamma probability distribution and a log link function to handle non‐normal residuals. For proportion values, a Gaussian distribution with an identity link function was used. For all models, treatments were used as a fixed effect and block as a random effect. Analysis of variance was done by type II sums of squares. In generalized models, the test of significance of variables was performed via analysis of deviance (Type II Wald Χ2‐tests, package car (Fox and Weisberg 2019)), while in linear mixed models, this was done via the F statistics estimated through Satterthwaite's approximation. The post hoc pairwise comparisons to assess differences between treatments were performed using the Tukey's method with the package emmeans (Lenth 2023). A significance threshold level of 0.05 was used for all statistical tests.

Principal component analysis (PCA) was performed on the 16 observations with the same fertilizer rate, using all root traits (centered and scaled). Treatment differences were assessed using a mixed‐effects model as above, with PC1 and PC2 scores as response variables. The ability of the PCA (i.e., the multivariate variability in root traits) to explain %MAOC was assessed with a linear mixed‐effects model as above, but with treatment as a random effect and PC1 as a fixed effect. We explored how individual root traits affected MAOC and POC using simple linear regressions separately for each trait.

## 3 Results

### 3.1 Aboveground Biomass, Root Biomass, and Rhizodeposition

The harvested biomass yield in the mixtures was up to three times higher than pure stand ryegrass with the same N fertilizer rate of 75 kg N ^−1^ha^−1^ year^−1^, and the highest yielding mixtures had similar yields as the high‐fertilized pure stand ryegrass receiving 300 kg N ^−1^ha^−1^ year^−1^ (Table S3). Aboveground yield from all three cuts combined was in total 14.3 t DM ha^−1^ in Mix2 and 19.7 and 19.2 t DM ha^−1^ in Mix6 and Mix18, respectively. The legume proportion ranged from 56% to 63% in the mixtures, with varying proportions of grass and forbs (Table S3). The proportion of N derived from the atmosphere in legume biomass ranged from 97% ± 1% to 99% ± 0.2% in the three mixtures (Table S3), showing that the amount of fertilizer N did not negatively affect N_2_ fixation in the legume component.

The root biomass concentration to a 25‐cm depth was highest in the six‐species mixtures (2.19 ± 0.16 g DM kg^−1^ dry soil) compared to all other treatments with the same fertilizer rate (*p* < 0.01, Figure S4h; Table S4), but similar to the high‐fertilized reference treatment Grass300N (2.25 ± 0.10 g DM kg^−1^ dry soil). Mix2 and Mix18 had the lowest root biomass (1.20 ± 0.11 and 1.33 ± 0.15 g DM kg^−1^ dry soil) while Grass75N had an intermediate root biomass (Table S4).

The quantity of net C rhizodeposition to 25 cm depth accumulated over the growing season was twice as high in pure stand ryegrass at the low N fertilizer rate (0.33 ± 0.02 g C kg^−1^ dry soil) compared to all mixtures, which had similar net rhizodeposition (ranging from 15 to 18 g C kg^−1^ dry soil) (Table S5). In contrast, rhizodeposition in the high‐fertilized monoculture grass was not significantly higher compared to the mixtures. We observed an inverse relationship between aboveground biomass yield and net rhizodeposition across all treatments at low N fertilizer application rate (χ^2^_1_ = 28.14, *p* < 0.001, Figure S5b). Similarly, a trend in the same direction was observed within treatments of monoculture perennial ryegrass with high and low N fertilization application rates, although more scattered and not statistically significant due to only a few observations (χ^2^_1_ = 2.69, *p* < 0.0.101; Figure S5b).

### 3.2 Treatment Differences in Morphological and Biochemical Root Traits

All mixtures had substantially lower root C:N ratios (*p* < 0.0001) and higher lignocellulose index (*p* < 0.0001) than the pure stand ryegrass treatments. In general, the mixtures had roots with a larger average diameter and lower SRL than the pure stand perennial ryegrass treatments (Table 2). Specific root length (SRL, [m root/g DM root]) and root length density (RLD, [cm root/cm^3^ of dry soil]) were almost twice as high in pure stand ryegrass treatments compared to the mixtures (*p* < 0.001, Table 2). The average root diameter (ARD, [mm]) was higher in mixtures compared to pure stand perennial ryegrass (*p* < 0.001, Table 2), with the six‐species mixture resulting in the highest ARD (Figure S4). Pure stand perennial ryegrass had higher root surface area (RSA, [cm^2^ root/g DM root]) than mixtures (*p* < 0.001, Table 2), while root tissue density (RTD, [g DM root/cm^3^ root]) was higher in mixtures compared to the pure stand grass treatments (*p* = 0.007, Table 2). Pairwise comparisons between individual treatments with the same low N fertilizer rate are given in Table 2.

**TABLE 2:** Root traits for the four different treatments with low N fertilizer amendment and the reference treatment pure stand grass with high N fertilizer rate.

Treatment	Specific Root Length (m g^−1^)	Root Length Density (cm cm^−3^ soil)	Average Root Diameter (mm)	Root Surface Area (cm^2^ g^−1^)	Root Tissue Density (g cm^−3^ root)	Root C:N ratio	Lignocellulose index in root biomass
*Grass300N*	*207 ± 11*	*61 ± 2*	*0.18 ± 0.00*	*1230 ± 60*	*0.16 ± 0.01*	*49.5 ± 1.9*	*0.09 ± 0.00*
Grass75N	216 ± 21 (c)	47 ± 9 (b)	0.18 ± 0.01 (a)	1310 ± 130 (c)	0.15 ± 0.02 (a)	55.6 ± 2.5 (a)	0.09 ± 0.00 (a)
Mix2	150 ± 14 (b)	25 ± 4 (a)	0.22 ± 0.01 (bc)	1010 ± 70 (bc)	0.19 ± 0.01 (ab)	24.6 ± 1.1 (b)	0.12 ± 0.00 (b)
Mix6	98 ± 9 (a)	28 ± 3 (a)	0.24 ± 0.01 (c)	730 ± 70 (a)	0.28 ± 0.03 (c)	23.9 ± 1.8 (b)	0.15 ± 0.01 (c)
Mix18	153 ± 15 (b)	26 ± 3 (a)	0.21 ± 0.01 (b)	990 ± 70 (b)	0.21 ± 0.01 (b)	28.2 ± 2.1 (b)	0.13 ± 0.01 (b)

*Note:* Compact letter display indicates statistical differences between individual treatments for each root trait, derived from post hoc comparisons where the high‐fertilized reference treatment (Grass300N) was not included in the models. Further visualization of treatment differences in root traits can be seen for all five treatments in Figure S4. See Table S2 for lignin, cellulose, and hemicellulose contents that are used to calculate the lignocellulose index. Treatments: Grass300N = perennial ryegrass with 300 kg N ha^−1^ year^−1^, Grass75N = perennial ryegrass with 75 kg N ha^−1^ year^−1^, Mix2 = perennial ryegrass and white clover, Mix6 and Mix18 = mixtures with 6 and 18 species (both containing grasses, legumes and forbs); see Table 1 for all species. All mixtures fertilized with 75 kg N ha^−1^ year^−1^.

### 3.3 Proportion of ^13^C Recovered in MAOC and POC Fractions

**FIGURE 1:**
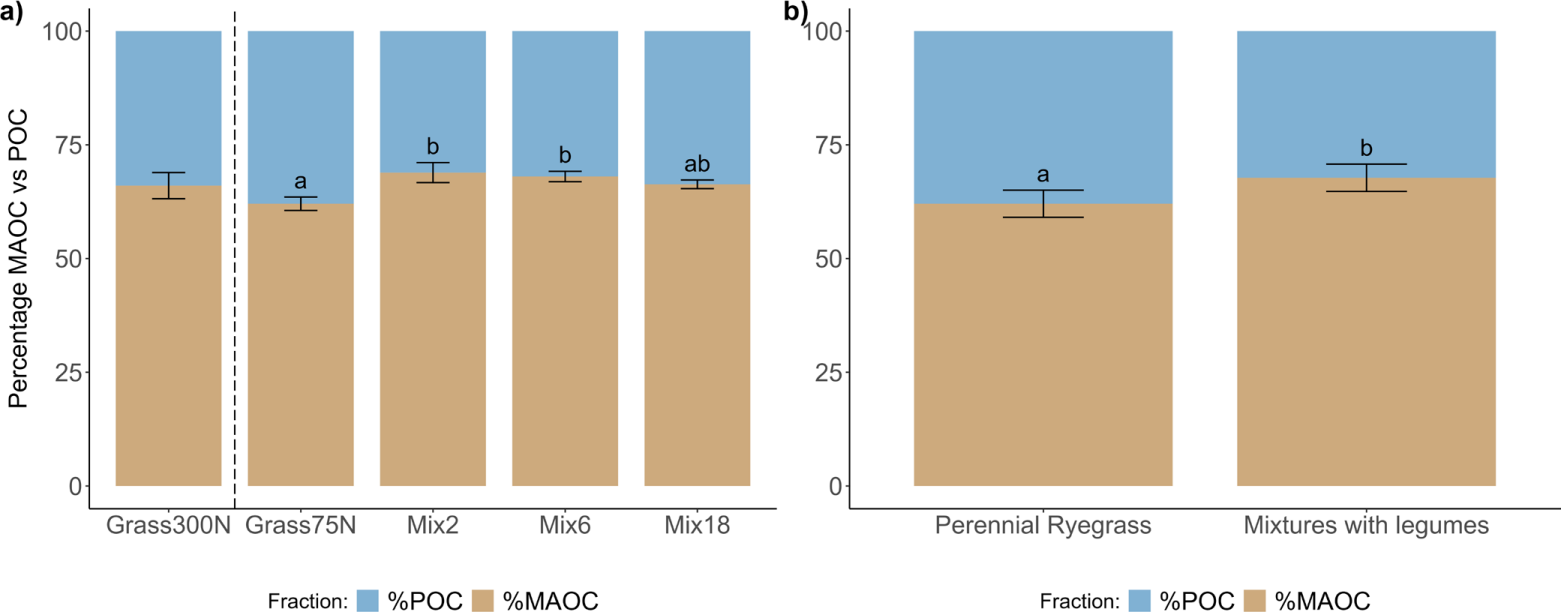
The relative distribution of ^13^C from recent rhizodeposition in MAOC and POC fractions for treatment comparisons (a) and by legume presence within low‐fertilized treatments (b). Treatments: Grass300N = Perennial ryegrass with 300 kg N ha^−1^ year^−1^, Grass75N = Perennial ryegrass with 75 kg N ha^−1^ year^−1^, Mix2 = perennial ryegrass and white clover, Mix6 and Mix18 = Mixtures with 6 and 18 species; see Table 1 and Table S1 for all species. All mixtures fertilized with 75 kg N ha^−1^ year.^−1^.

The proportion of recently formed MAOC vs. POC (%MAOC and %POC) differed significantly between the treatments (Figure 1a), where the %MAOC was significantly higher in mixtures including legumes compared to monoculture ryegrass with the same amount of fertilizer (*p* = 0.006, Figure 1b). In all treatments, more than 60% of the ^13^C was recovered in the MAOC fraction (range 62%–69%). No significant differences occurred when adding red clover and forbs (Mix6 and Mix18) compared to Mix2 (Figure 1a). Mix2 (*p* < 0.001) and Mix6 (*p* = 0.004) had approximately 6% higher %MAOC than Grass75N, while a strong tendency to higher %MAOC was found in Mix18 (*p* = 0.066) compared to Grass75N (Table S5). The Grass300N reference showed an intermediate %MAOC with higher variance and was not significantly different from any of the other treatments.

### 3.4 Quantity of MAOC and POC Fractions

**FIGURE 2:**
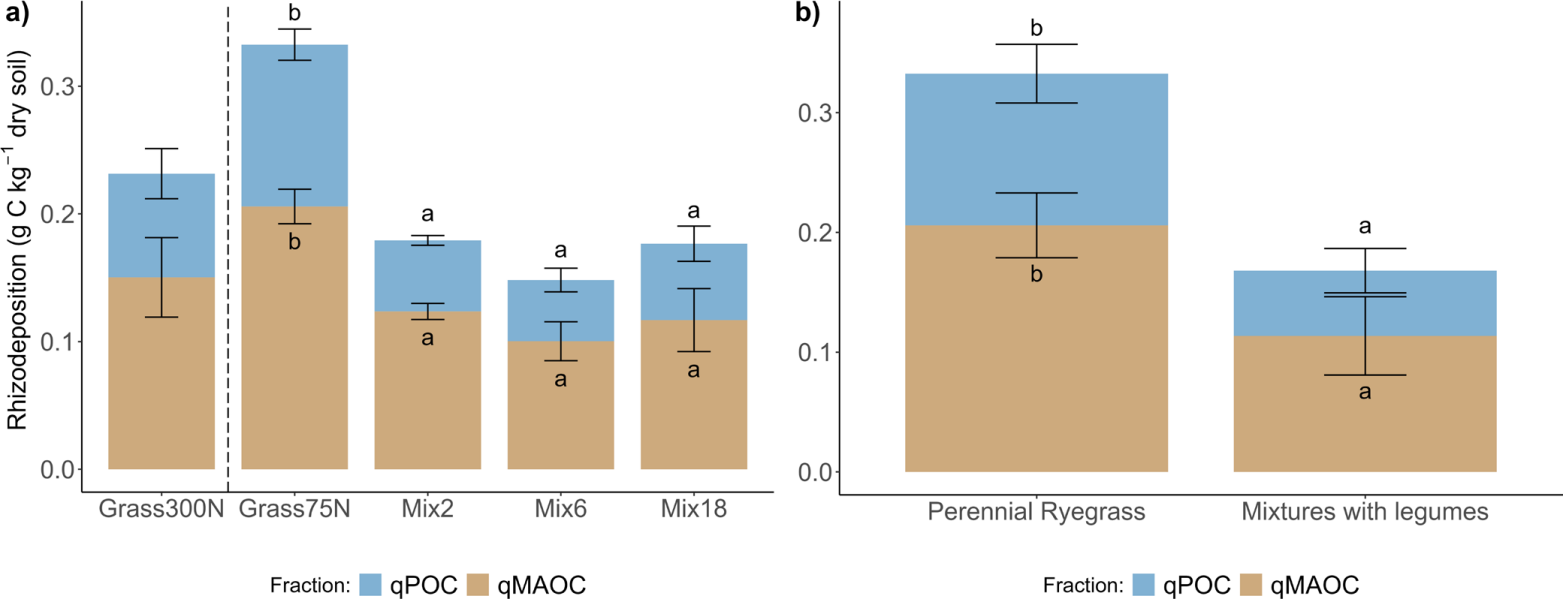
Differences in the quantities of MAOC and POC (qMAOC and qPOC) of total rhizodeposition based on recovered ^13^C tracer in each fraction for treatment comparisons (a) and by legume presence within low‐fertilized treatments (b). Treatments: Grass300N = Perennial ryegrass with 300 kg N ha^−1^ year^−1^, Grass75N = Perennial ryegrass with 75 kg N ha^−1^ year^−1^, Mix2 = perennial ryegrass and white clover, Mix6 and Mix18 = Mixtures with 6 and 18 species; see Table 1 and Table S1 for all species. All mixtures fertilized with 75 kg N ha^−1^ year^−1^.

While the proportion of MAOC was higher in mixtures including legumes, the quantities of both MAOC and POC (qMAOC and qPOC) of total rhizodeposition were higher in monoculture ryegrass compared to mixtures with legumes (*p* < 0.001, Figure 2). These results were driven by the substantially higher total net C rhizodeposition in pure stand perennial ryegrass (Grass75N) compared to mixtures with legumes (Table S5).

The qMAOC was significantly higher in Grass75N compared to Mix2 (*p* = 0.002), Mix6 (*p* = 0.003), and Mix18 (*p* = 0.047). The differences were even stronger for qPOC when comparing Grass75N to Mix2 (*p* < 0.001), Mix6 (*p* = 0.002) and Mix18 (*p* = 0.018), due to the higher %POC in Grass75N compared to mixtures. Comparing Grass75N with Grass300N, there was no significant effect of N fertilizer on the quantity of MAOC (*p* = 0.201) or POC (*p* = 0.144), and despite higher average qMAOC and qPOC in the high‐fertilized ryegrass compared to the mixtures, the differences were not significant.

### 3.5 Root Traits in Relation to the Quantity of MAOC and POC

**FIGURE 3:**
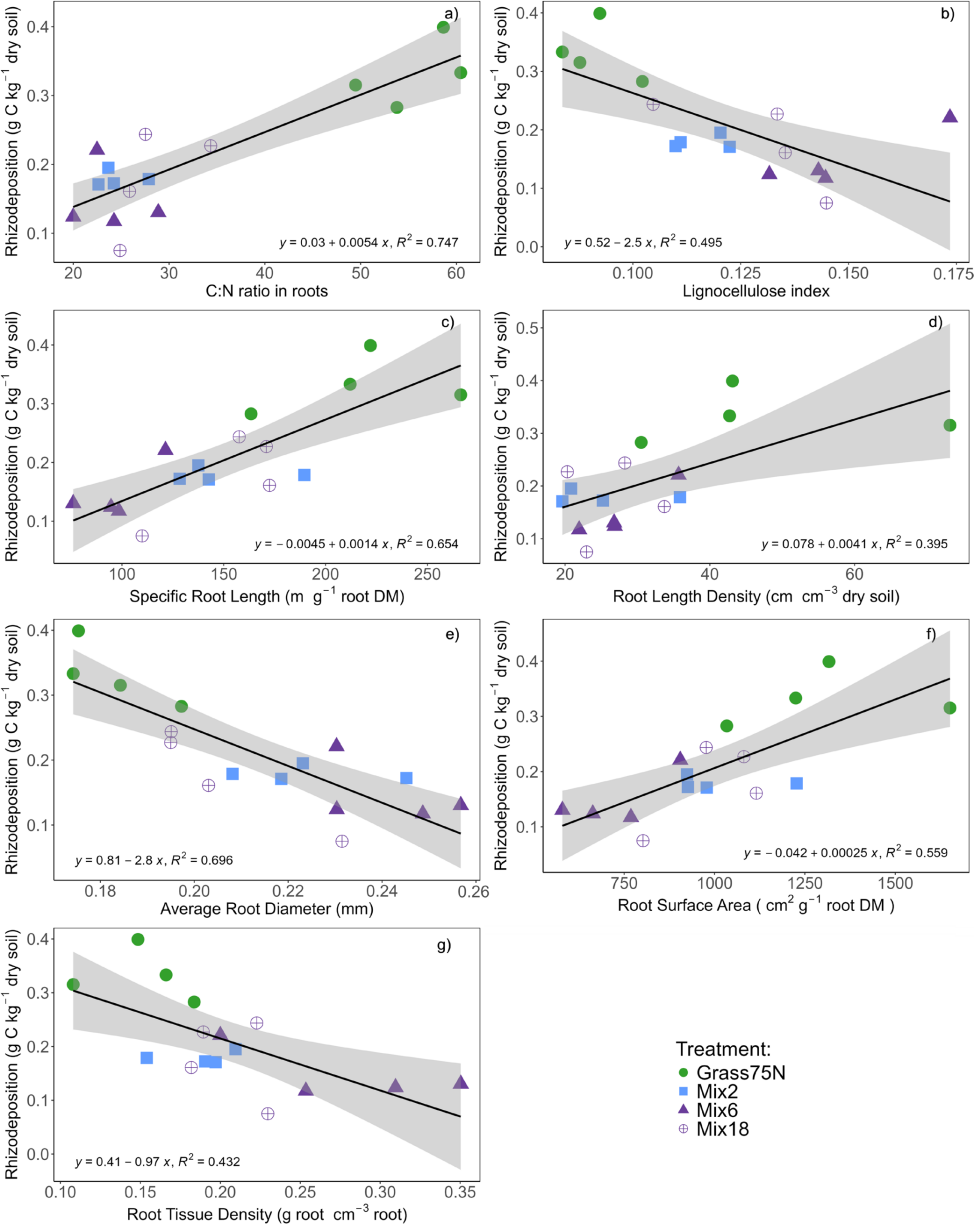
Linear regression models with individual root traits (panel a‐g) and the quantity of C rhizodeposition. The linear regression models with quantity of MAOC and POC, respectively (Figure S6), were driven by the total amount of C rhizodeposition, and thus the two fractions were both well represented by the overall regressions with total C rhizodeposition. Treatments: Grass75N = Perennial ryegrass with 75 kg N ha^−1^ year^−1^, Mix2 = perennial ryegrass and white clover, Mix6 and Mix18 = Mixtures with 6 and 18 species; see Table S1 for all species. All mixtures fertilized with 75 kg N ha^−1^ year.^−1^.

Total root biomass did not show any relationship with the quantity of MAOC (*F*_1,14_ = 0.589, *p* = 0.456, *R* ^2^ = 0.04), the quantity of POC (*F*_1,14_ = 0.042, *p* = 0.841, *R* ^2^ = 0.003) or the total quantity of C rhizodeposition (*F*_1,14_ = 0.282, *p* = 0.604, *R* ^2^ = 0.02). However, individual root traits were strongly related to the quantity of MAOC, POC, and total C rhizodeposition (Figure 3; Figure S6). Since both qMAOC and qPOC were driven by the total amount of rhizodeposition, all the relationships between the root traits and either qMAOC or qPOC (Figure S6) were well represented by the relationships with the sum of both qMAOC and qPOC, i.e., the total quantity of C rhizodeposition (Figure 3). Higher SRL, RLD, RSA, and root C:N ratio were associated with higher C rhizodeposition, while ARD, RTD, and the lignocellulose index were associated with lower C rhizodeposition (Figure 3).

### 3.6 Root Traits in Relation to the Relative Proportion of MAOC and POC

#### 3.6.1 Linear Regressions

**FIGURE 4:**
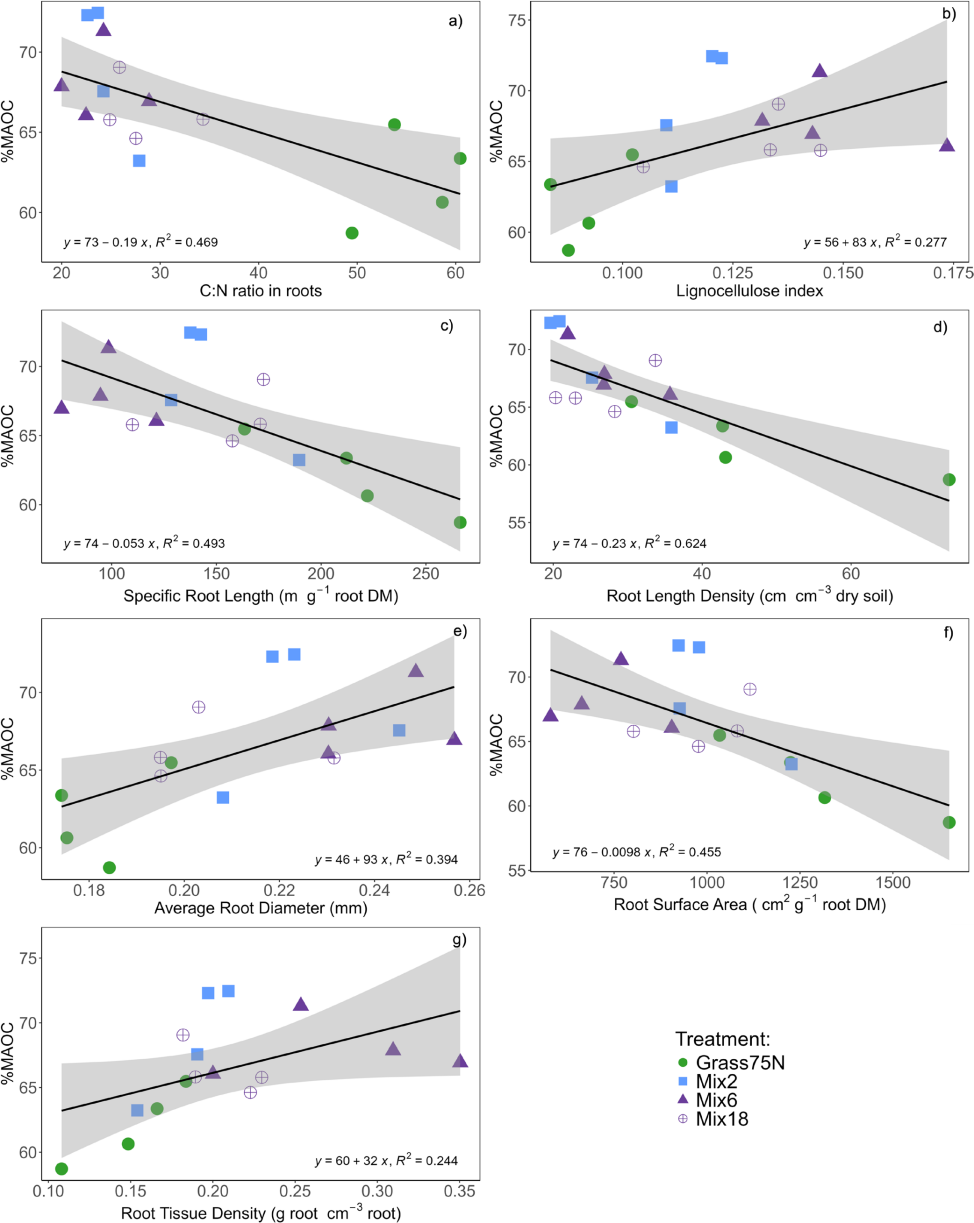
Linear regression models with individual root traits (panel a‐g) and relative proportion of MAOC (%MAOC). Treatments: Grass75N = Perennial ryegrass with 75 kg N ha^−1^ year^−1^, Mix2 = perennial ryegrass and white clover, Mix6 and Mix18 = Mixtures with 6 and 18 species; see Table S1 for all species. All mixtures fertilized with 75 kg N ha^−1^ year^−1^.

When tested individually, all biochemical and morphological root traits showed significant relationships with %MAOC (and inversely with %POC). A higher root C:N ratio was associated with lower %MAOC (*F*_1,14_ = 14.09, *p* = 0.003; Figure 4a). Conversely, a higher lignocellulose index was associated with higher %MAOC (*F*_1,14_ = 5.36, *p* = 0.036, Figure 4b).

Higher SRL (*F*_1,14_ = 13.62, *p* = 0.002), RLD (*F*_1,14_ = 23.28, *p* < 0.001) and RSA (*F*_1,14_ = 11.71, *p* = 0.004) were negatively associated with %MAOC (Figure 4). Thus, %POC was higher for plant communities with high SRL, RLD, and RSA, three traits that were higher in the pure stand ryegrass treatments compared to the mixtures. On the other hand, a higher ARD (*F*_1,14_ = 9.22, *p* = 0.009) and RTD (*F*_1,14_ = 4.74, *p* = 0.049) were positively related to %MAOC (Figure 4). There was no relationship between total root biomass and %MAOC (*F*_1,14_ = 0.72, *p* = 0.411).

Comparing Grass75N (low‐fertilized pure stand ryegrass) to the reference treatment Grass300N (high‐yielding, high‐fertilized pure stand ryegrass), most root traits were similar except for the root C:N ratio, which was significantly lower in Grass300N compared to Grass75N (*p* = 0.023). When testing the linear relationships between the individual root traits and %MAOC, these were all weakened if including the reference treatment Grass300N, as this treatment showed contrasting relationships with %MAOC compared to the treatments with the low fertilizer rate (Figure S7). Further, Grass300N showed a substantially higher variation between replicates than Grass75N.

#### 3.6.2 PCA With Root Traits and the Relative Proportion of MAOC

**FIGURE 5:**
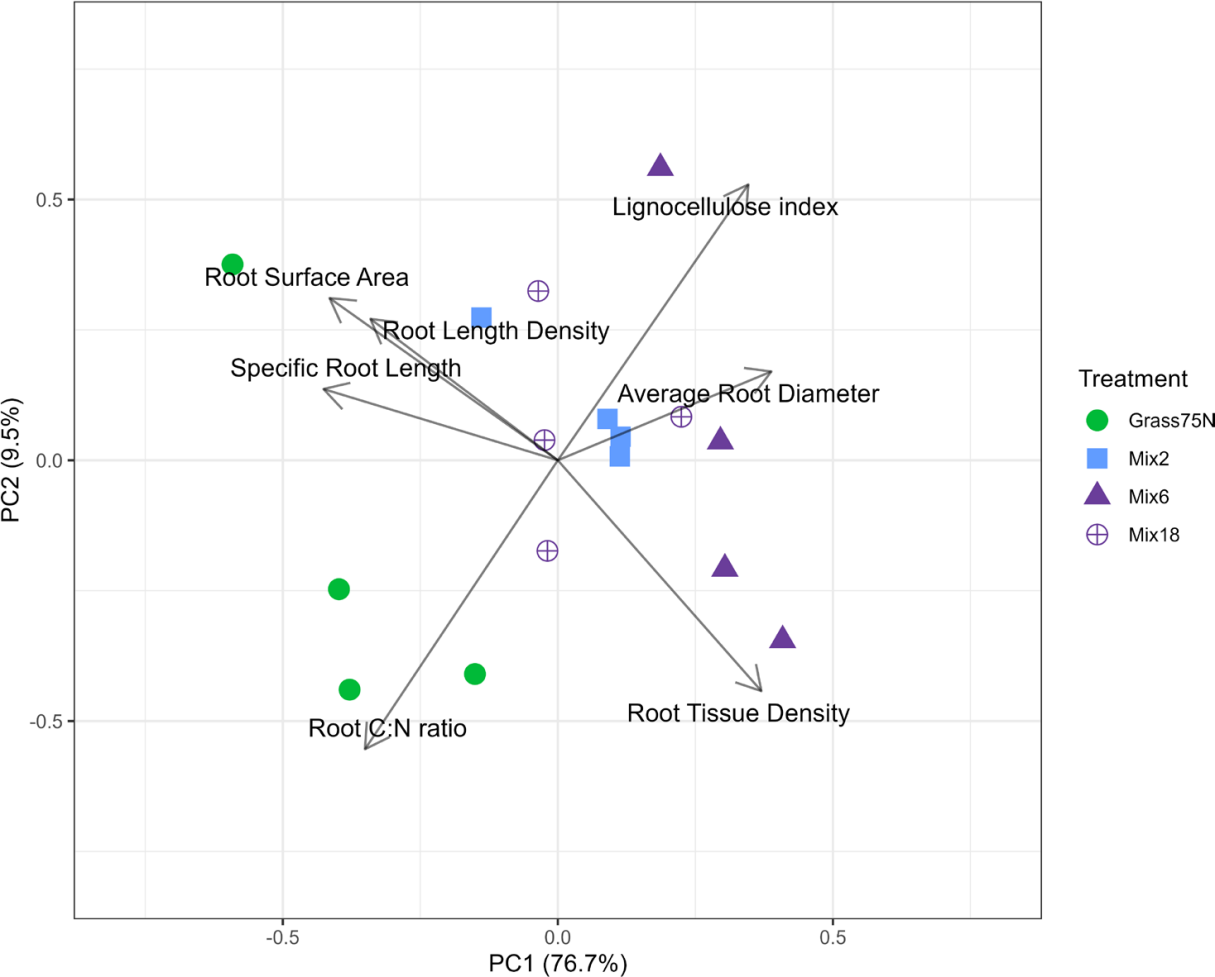
Principal Component Analysis (PCA) with all root traits. The treatments as indicated by symbols in the PCA are: Grass75N = Perennial ryegrass with 75 kg N ha^−1^ year^−1^, Mix2 = Perennial ryegrass and white clover, and Mix6 and Mix18 = mixtures with 6 and 18 species; see Table S1 for all species.

**FIGURE 6:**
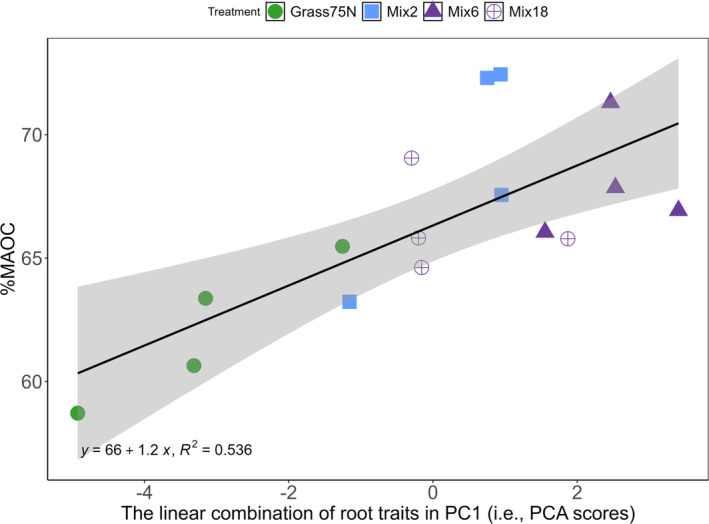
A linear regression between the combination of root traits in PC1 from the PCA (Figure 5) and the proportion of mineral‐associated organic carbon (%MAOC) showed a positive slope (χ^2^_1_ = 17.79, *p* < 0.001) toward higher %MAOC in the mixtures compared to monoculture perennial ryegrass (Grass75N).

We carried out a PCA to evaluate the joint variability in root traits. The combinations of PC1 and PC2 explained 86.2% of the variation, with the majority on PC1. SRL and especially RLD were strongly associated with RSA (Figure 5). Higher values of these three morphological traits and particularly for the root C:N ratio were linked to the pure stand ryegrass. ARD, RTD, and the lignocellulose index were positively associated with the mixtures (Figure 5). The linear combination of root traits in PC1 (i.e., the PCA scores) showed a significant positive relationship with %MAOC (χ^2^_1_ = 17.79, *p* < 0.001) when tested in a linear mixed‐effects model (Figure 6). Due to the root traits differing between treatments, this association relates to higher %MAOC in the mixtures compared to monoculture perennial ryegrass (Grass75N) (Figure 6). When testing the differences between treatments with PC1 scores as the response variable (explaining 76.7% of the variability), Grass75N was significantly different from all 3 mixtures (*p* < 0.001 for Mix6 and *p* < 0.01 for Mix2 and Mix18) with no differences between mixtures. The difference between mixtures and monoculture grass illustrates the potential to modify root traits and associated changes in %MAOC using plant mixtures.

If %MAOC was included in the PCA as a variable together with the root traits (Figure S8), the two‐species mixtures with perennial ryegrass and white clover had the strongest association with %MAOC of all treatments.

## 4 Discussion

### 4.1 Grasses Increase the Quantity of Both MAOC and POC

The amount of C rhizodeposition was twice as high in low‐fertilized pure stand ryegrass compared to the species mixtures including legumes. Thus, the quantity of both MAOC and POC fractions was also significantly higher in pure stand ryegrass compared to the mixtures, as these were driven by the quantity of rhizodeposition rather than by the relative proportion of MAOC compared to POC. This underlines the importance of input quantity instead of quality as a key driver of MAOC formation (Angst et al. 2023; Begill et al. 2023). The high level of rhizodeposition in the low‐fertilized perennial grass can be linked to high belowground C investments to overcome nutrient shortage (Bais et al. 2006; Jones et al. 2009; Rasmann and Turlings 2016). However, both the low‐ and high‐fertilized pure stand perennial ryegrass had high rhizodeposition, which indicates that grasses play a key role for C inputs via rhizodeposition quantity—and thus in MAOC formation. Although differences in root traits occur between grass species (Barneze et al. 2024; Picon‐Cochard et al. 2011), perennial ryegrass can be considered representative of the grass functional group, as it has intermediate values for most root traits such as RLD, SRL, and root N content when compared to other grass species (Fort et al. 2012; Rossi et al. 2020). Consistent with this, perennial ryegrass shows distinctively different root traits compared to forbs and legumes, e.g., in terms of SRL, RLD, and N content (Barneze et al. 2024; Gould et al. 2016; Sweeney et al. 2021; Williams et al. 2021). Therefore, our results are likely to be applicable for other grasses than just perennial ryegrass, although dedicated studies will need to confirm this.

The relationships between individual root traits and the quantity of MAOC and POC were similar. Accordingly, the regressions with both MAOC and POC were well represented by the total amount of C rhizodeposition. The similar response of MAOC and POC fractions could be due to the role and large pool of small particulates and root hairs in facilitating the rapid turnover of new OM and thus the formation of MAOC (Angst et al. 2021; Villarino et al. 2021; Witzgall et al. 2021).

The positive relationship observed between RSA and the quantity of C rhizodeposition (i.e., qMAOC + qPOC) confirmed our hypothesis (1) and is in line with the results of Engedal et al. (2023). However, our study reveals that other root traits (i.e., root C:N ratio and RLD) are better predictors of total C rhizodeposition and %MAOC than RSA. Further, the RSA was driven by root length rather than diameter, and thus the treatment with the highest SRL (i.e., Grass75N) also showed the highest quantity of MAOC and POC (Table S5). The increased amount of rhizodeposition with high SRL and surface area explains why grasses can stimulate higher C input to soil compared to other functional groups (Bardgett et al. 2014; Deyn et al. 2008; Henneron et al. 2019), and it also suggests that identifying legume and forb species with these traits can increase the amount of rhizodeposition in mixtures.

### 4.2 Legumes—Not Species Richness—Increase the Proportion of MAOC

The relative proportion of ^13^C recovered in the MAOC (%MAOC) was not increased by species richness. While %MAOC was higher for Mix2 and Mix6 than for pure stand perennial ryegrass, there were no differences between the 2, 6, or 18 species mixtures. In fact, although not statistically significant, the average %MAOC decreased from 2 to 6 and from 6 to 18 species, in contrast to our hypothesis (2). Despite earlier studies reporting that higher plant species diversity leads to higher diversity in rhizodeposits (Eisenhauer et al. 2017; Lange et al. 2019), this did not translate to higher %MAOC in our study. Instead, our results with similar expressed legume proportions suggest functional group and legume identity to be driving %MAOC.

Mixtures with legumes showed up to 6% higher %MAOC than pure stand ryegrass with the same low fertilizer rate, in line with hypothesis (3). Correspondingly, we found that a lower root C:N ratio typical of legumes, here considered a proxy of root rhizodeposition quality, increased %MAOC. This is in accordance with the concept of increased residue quality (high N content) leading to higher MAOC formation efficiency (Cotrufo et al. 2013; Engedal et al. 2023; Villarino et al. 2021; Zhang et al. 2022). The N_2_‐fixing legumes have higher N concentration in their biomass compared to non‐legumes, which increases the N availability for the soil microbial community through exudation of N‐rich compounds like amino acids (Czaban et al. 2016; Høgh‐Jensen and Schjoerring 2001; Lesuffleur et al. 2012). This ensures a combination of easily accessible C and N in favor of in vivo turnover (Liang et al. 2017) and thus facilitates a higher potential microbial stabilization (Peixoto et al. 2022).

Including forbs and red clover in the mixtures (i.e., Mix6 and Mix18) slightly decreased %MAOC compared to the two‐species grass‐legume mixture with white clover (Table S5). This indicates that the addition of red clover and forbs to the mixture dilutes the positive effect of white clover on the %MAOC, which was also shown by the PCA when %MAOC was included together with the root traits (Figure S8). Previous studies have shown a hierarchy of N generosity among grassland species with differences in the degree to which species donate N to companions (white clover>red clover > > forbs/non‐legumes) (Pirhofer‐Walzl et al. 2012; Rasmussen et al. 2012), which was similar to the hierarchy we found in terms of %MAOC in our present study. Importantly, although the 6 and 18 species mixtures consisted of one more functional group than the two‐species mixtures (i.e., forbs), the realized proportion of legume biomass was similar for all three mixtures (Table S3) with red clover markedly dominating the Mix6 and Mix18 swards. Consequently, the decrease in %MAOC cannot be explained by the inclusion of forbs, but rather by a shift in legume identity. Furthermore, mixtures with legumes also showed a higher lignocellulose index in root biomass than pure stand perennial ryegrass. While this was not surprising in itself, the lignocellulose index was positively related to %MAOC in contrast to our expectations. However, the increased %MAOC in legumes may likely be due to other legume traits (e.g., C:N ratio) that expressed stronger relationships. More observations in a narrow range of species are needed to investigate the direct effect of lignin and the lignocellulose index on %MAOC.

### 4.3 Root Traits Are Strong Predictors of the Proportion of MAOC

The strong relationship between the linear combination of all root traits (PC1 scores) and %MAOC (Figure 6) demonstrates the potential to modulate the fate of C rhizodeposition into distinct SOC pools using trait‐based species selection for mixture design. The individual root traits revealed significant linear relationships with %MAOC; however, the direction of these relationships was inverse compared to the relationships with the total quantity of C rhizodeposition (and qMAOC and qPOC). Higher SRL and RLD in pure stand ryegrass were associated with higher %POC, in accordance with our hypothesis (1) that the more fibrous and fine roots of perennial ryegrass would translate into more POC. This could be due to more root biomass physically protected in aggregates (Bardgett et al. 2014). Also, in accordance with hypothesis (1), a higher ARD was associated with higher %MAOC. This can possibly be explained by higher AMF colonization facilitated by large root diameter (Sweeney et al. 2021). Increased AMF may increase the formation efficiency of microbial‐derived MAOC by increased rhizodeposition (Zhou et al. 2020) and increase the physical protection of SOC by networks of hyphae facilitating the formation of stable soil aggregates (Bardgett et al. 2014; Hallett et al. 2008; Poirier et al. 2018), which in turn can increase the formation of both microbial‐ and plant‐derived POC (Amelung et al. 2023; Liang et al. 2017; Mueller et al. 2024).

RSA was found to increase %POC, not %MAOC. This is surprising because increased interaction between the root surface and mineral surfaces is generally found to alter MAOC (Poirier et al. 2018; Sokol and Bradford 2019; Sokol et al. 2022), and a higher MAOC formation efficiency has been observed for species with fine root systems compared to species with coarser roots by Engedal et al. (2023) who used the amount of soil adhering to roots (i.e., rhizosphere soil) as a proxy for RSA. The reason may be that RSA across the plant communities tested in our experiment was driven by the higher total root length observed in the grass treatments, which overruled the effects of a higher ARD in the mixtures. The positive effect of fine roots on %POC in pure stand grass (e.g., via aggregation) may have been stronger than potential positive effects of fine roots and high RSA on %MAOC, e.g., via increased contact between the soil microbiome and rhizodeposition point of entry (Sokol and Bradford 2019). In support of this, the association of low C:N ratio with legume mixtures and %MAOC was more important than that of RSA and %MAOC. Higher RTD increased %MAOC, which was unexpected, considering that a high RTD is generally associated with low decomposition rates due to higher toughness and strength of the roots (Goebel et al. 2011; Makita et al. 2015). As with RSA, such an effect of RTD may have been overruled by the strong effect of low root C:N ratio in legume rhizodeposits, which have been shown to promote greater bacterial biomass than rhizodeposits from grass (Kušlienė et al. 2014).

While clear differences between treatments were observed in all morphological root traits (Figure S4), and despite significant relationships with all these traits and %MAOC when tested individually, the strongest relationships were negative associations between %MAOC and root C:N ratios and RLD (Figure 4). This was confirmed by a PCA where %MAOC was included together with all root traits (Figure S8), where RLD was strongly positively associated with pure stand perennial ryegrass and %POC (inverse for %MAOC), while a low C:N ratio was associated with mixtures and high %MAOC.

Aboveground biomass yield and C released to the soil via root exudates are often negatively related (e.g., Chen et al. 2022; Nguyen 2003; Pausch and Kuzyakov 2018), with different mechanisms contributing to explain this relationship. The increased rate of root exudates under nutrient‐poor conditions can be driven by the plant's need to increase nutrient uptake via plant–soil–microbial interactions (Canarini et al. 2019; Jones et al. 2009; Selosse and Rousset 2011). However, Prescott et al. (2020) argue that the release of C from plants to soil (e.g., via root exudates) under nutrient constraints is primarily driven by the need to reduce the level of surplus photosynthates in leaves to avoid photo‐damage (the surplus C hypothesis). In our study, we observed such an overall negative relationship between yield and rhizodeposition when yield increased due to the inclusion of legumes (Figure S5a). This negative relationship could also be observed when yield increased due to fertilization in monoculture ryegrass, but in this case, the trend was less evident, possibly due to the lower data availability (Figure S5b). However, as our study shows, other factors are at play at the species level. Plant species differ in terms of rhizodeposition rate due to specific root traits, and the relationship between rhizodeposition and yield differs between mixtures with legumes (Henneron et al. 2019; Pirhofer‐Walzl et al. 2012; Rasmussen et al. 2012). Consequently, and in line with the results presented in this study, species‐specific traits in mixtures can regulate C rhizodeposition quantity and quality on top of mere yield and surplus C effects. Elucidating these species‐specific effects further requires a dedicated experimental setup targeting specific effects of each individual species.

### 4.4 Designing Grassland Mixtures to Enhance the Total Quantity of MAOC

Grasslands build SOM both through rhizodeposition from the living plant and through eventual turnover of the standing root biomass either during the subsequent growing season or upon termination. Among all treatments with similarly low N fertilizer, root biomass was highest in the six‐species mixture (Table S4) which contained red clover and chicory, species with thick taproots and presumably lower rhizodeposition in topsoil (Black et al. 2009; Pirhofer‐Walzl et al. 2012; Mortensen, Abalos, Rasmussen 2025). In contrast, rhizodeposition and therefore the quantity of both MAOC and POC, was enhanced by perennial ryegrass, as shown in the present study. The only treatment that showed similar root biomass to Mix6 was the high‐fertilized reference grass treatment (Lp300N) (Table S4). Thus, to obtain both high root C and increase the amount of rhizodeposition and the relative proportion of MAOC at the same time, sufficiently high grass proportions seem to be needed in legume‐based mixtures. In our case, the realized proportion of legumes ranged, on average, from 56% to 63% for the three mixtures (Table S3). Hence, in terms of the quantity of both MAOC and POC, the mixtures may potentially have benefitted from a more competitive grass companion, or less competitive legumes/forbs, to increase the total amount of rhizodeposition, which in turn could be more effectively formed into MAOC by fungi and bacteria with easily accessible N from the legumes.

High fertilization of the pure stand perennial ryegrass reduced rhizodeposition, but this treatment still maintained an average above all the mixtures. Thus, the higher level of rhizodeposition in the pure stand grass treatment(s) indicates that a high presence of grass in multi‐species mixtures with legumes could combine a high quantity of rhizodeposition (grass component) with increased %MAOC (legume component). Besides species choice and sown proportions, N availability is the key to regulating legume vs. non‐legume presence in intensively managed mixed swards (Suter et al. 2015). Our results indicate that N fertilization of 75 kg N ha^−1^ year^−1^ might be low enough to allow legumes to establish successfully and fix atmospheric N at high rates (> 97%Ndfa), while providing grasses with sufficient mineral N to achieve satisfactory yields for productive systems comparable to those receiving much higher N fertilizer inputs. Future studies could evaluate the level and type of N fertilization in legume‐based mixtures to further optimize yield and soil C storage, also considering the overall GHG emissions related to the production of N fertilizer and to soil N_2_O emissions (Abalos et al. 2019; Chojnacka et al. 2019).

### 4.5 Methodological Considerations

The fractionation protocol we used has the advantage that it is relatively simple and thus practical to align with other studies of SOC fractionation. However, it does not differentiate between occluded POC and free POC (Golchin et al. 1994; Kölbl and Kögel‐Knabner 2004; Mueller and Koegel‐Knabner 2009), which could have given insight into how the mixtures and pure stand perennial ryegrass may differ in these two groups of POC. With soil dispersion and size fractionation, there is also a risk that a part of the POC is below 50 μm and thus interpreted as MAOC if traveled through the 50‐μm sieve. However, our analysis of the C:N ratio in soil fractions shows clear and qualitative differences between the fractions, which indicate that mixing between the fractions has been limited. Further, our fractionation protocol did not account for differences in dissolved organic carbon (DOC), but as this pool is relatively small and in equilibrium with the other C pools (Lavallee et al. 2020), we do not expect potential differences between treatments to be of importance for the overall effects on soil C storage.

The effect of species mixtures on the overall soil C balance depends on both C inputs and outputs. In this study, we only investigated the effect of species mixtures and root traits on the C input side. For a full overview of the effects of grassland species and root traits on soil C storage, potential species effects on the turnover of existing SOM (i.e., the priming effect) need to be taken into account (Huo et al. 2017; Lloyd et al. 2016), with a different experimental setup than ours. The POM fraction may be more vulnerable to a potential priming effect than the MAOM fraction (Villarino et al. 2021). Furthermore, since only perennial ryegrass was present at all species richness levels, our study did not cover potential sampling effects, and we cannot rule out identity effects of certain species that were only present in the multi‐species mixtures (Loreau and Hector 2001; Zuppinger‐Dingley et al. 2014). Fully disentangling this aspect would require an extensive study with all species in monocultures.

In the present study, we only investigated the fate of rhizodeposition into MAOC and POC fractions in the topsoil to 25 cm depth. Although the majority of plant belowground C input enters the topsoil, grassland mixtures differ in their distribution of roots and rhizodeposition in subsoil layers (Mortensen, Abalos, Rasmussen 2025), and root fragments and rhizodeposition constitute an important part of subsoil C input, and their contribution to total plant C input increases with depth (Liang et al. 2022). Therefore, it is possible that mixtures with deep‐rooted species such as red clover and forbs (i.e., Mix6 and Mix18) that have a larger overall C investment in subsoil than grasses, on the longer term, when the roots decompose, would add more both qMAOC and qPOC in deeper soil layers than pure stand grass. Further, the longer‐term fate of the living root biomass at the time of sampling was not included in the present study, and this could be critical as mixtures could be expected to have higher POC and MAOC formation from coarser root turnover with a higher lignocellulose index on the longer term. Accounting for these two aspects would demand a study that extends further in depth and time than our present study.

## 5 Implications

We were able to demonstrate the importance of legumes with a low root C:N ratio for the formation of MAOC from rhizodeposition in intensively managed grassland mixtures. On the contrary, perennial ryegrass with a high root length and surface area was especially important for the formation of POC and showed higher total rhizodeposition. Furthermore, we clearly showed that the total amounts of rhizodeposition‐derived C are the main driver for the quantity of both major soil C pools, POC and MAOC.

While the formation of both MAOC and POC is crucial for the modulation of the persistence of the overall soil C pool, the importance of increasing each pool for soil C storage depends on the specific soil texture and soil C status of a given site (Angst et al. 2023). Therefore, we suggest that the species composition of grassland mixtures can be fine‐tuned according to site‐specific conditions to optimize the persistence of rhizodeposition‐derived C as a climate change mitigation tool, while securing high and stable yield with lower N fertilizer inputs. Our results present a framework to design such mixtures, which need to be validated under various pedoclimatic conditions.

Our study reveals that plant communities in agricultural systems can be designed following a trait‐based approach to steer the proportion of rhizodeposited C allocated into soil C pools of differing bioavailability and thus persistence. By unraveling the traits behind the relationships between plant communities and MAOC and POC, we propose a guide for species and cultivar selection for field experiments and on‐farm applications beyond the species tested in our study.

## Author Contributions

**Esben Øster Mortensen:** conceptualization, data curation, formal analysis, investigation, methodology, visualization, writing – original draft, writing – review and editing. **Diego Abalos:** conceptualization, methodology, validation, visualization, writing – review and editing. **Tine Engedal:** investigation, methodology, validation, writing – review and editing. **August Kau Lægsgaard:** data curation, writing – review and editing. **Kirsten Enggrob:** data curation, writing – review and editing. **Carsten W. Mueller:** conceptualization, methodology, validation, writing – review and editing. **Jim Rasmussen:** conceptualization, funding acquisition, project administration, validation, visualization, writing – review and editing.

## Conflicts of Interest

The authors declare no conflicts of interest.

## Supporting information


Data S1.

## Data Availability

The data that support the findings of this study are openly available in the Zenodo repository at https://doi.org/10.5281/zenodo.14917866.
