# Supplementary material for: Smart Mixture Design Can Steer the Fate of Root‐Derived Carbon Into Mineral‐Associated and Particulate Organic Matter in Intensively Managed Grasslands
Source: Glob Chang Biol. 2025 Mar 6;31(3):e70117. doi: 10.1111/gcb.70117 (PMC11883481; doi:10.1111/gcb.70117)
Supplement: Supplementary file 1 — Data S1. [file GCB-31-e70117-s001.docx]

**Supplementary material**

***Smart mixture design can steer the fate of root derived carbon into mineral-associated and particulate organic matter in intensively managed grasslands***

Esben Øster Mortensen^1,5,^*, Diego Abalos^1,6^, Tine Engedal^2^, August Kau Lægsgaard^1^, Kirsten Enggrob^1^, Carsten W. Mueller^3, 4^, and Jim Rasmussen^1^

^1^Department of Agroecology, Aarhus University, 8830 Tjele, Denmark

^2^Department of Plant and Environmental Sciences, University of Copenhagen, 1871 Frederiksberg, Denmark

^3^Institute of Ecology, Chair of Soil Science, Technische Universität Berlin, Berlin, Germany

^4^Department of Geosciences and Natural Resource Managements, University of Copenhagen, Copenhagen, Denmark

^5^CBIO Aarhus University Centre for Circular Bioeconomy, Aarhus University, 8830 Tjele, Denmark

^6^iCLIMATE Interdisciplinary Centre for Climate Change, Aarhus University, 4000 Roskilde, Denmark

*Corresponding author: Esben Øster Mortensen, eom@agro.au.dk

**Journal:** Global Change Biology

Table S1: Treatments consisted of: Pure stand ryegrass with two levels of fertilizer, and mixtures with sown species composition based on number of seeds. In mixtures, the legume component represented 25%, based on agronomic recommendation by the commercial seed provider.

| **Management** | | |  | **Sown species composition** | | | | | | |
| --- | --- | --- | --- | --- | --- | --- | --- | --- | --- | --- |
| **Treatment** | **N fertilizer (kg N ha^-1^ yr^-1^)** §2 | **% sown legume** | Common name: | Perennial Ryegrass | Tall fescue | Plantain | Chicory | White Clover | Red Clover | Additional species in Mix18, see note |
|  |  |  | *Latin name:* | *Lolium perenne* | *Festuca arundinacea* | *Plantago lanceolata* | *Cichorium intybus* | *Trifolium repens* | *Trifolium pratense* |  |
|  |  |  | *Variety:* | *Arsenal* | *Swaj* | *-* | *Spadona* | *Munida* | *Larus* |  |
| **Grass75N** | **75** | **0** |  | 100 |  |  |  |  |  |  |
| **Grass300N** | **300** | **0** |  | 100 |  |  |  |  |  |  |
| **Mix2** | **75** | **25** |  | 75 |  |  |  | 25 |  |  |
| **Mix6** | **75** | **25** |  | 20 | 20 | 18 | 17 | 19 | 6 |  |
| **Mix18** §1 | **75** | **25** |  | 8.8 | 7.3 | 5.5 | 5.2 | 4.2 | 2.6 | 66.4 |

Note §1: The 66.4 % additional species in Mix18 were the following. Grasses: Timothy (18.3 %, Phleum pretense, var. Presto), Cocksfoot (6.7 %, Dactylis glomerata, var. Donata), Meadow fescue (5.1 %, Festuca pratensis, var. Liherold), and Hybrid ryegrass (4.1%, Lolium hybridum, var. AberEcho). Legumes: Alsike clover (4.2 %, Trifolium hybridum, var. Aurora), Bird’s-foot trefoil (3.7 %, Lotus corniculatus, var. Leo), Lucerne (2.9 %, Medicago sativa, var. SW Nexus), Black medick (2.9 %, Medicago lupulina, var. Virgo), Crimson clover (2.5 % Trifolium incarnatum, var. Heusers Ostsaat), and Serradella (2.2 % Ornithopus sativus). Forbs: Caraway (4.7 %, Carum carvi, var. Rekord) and Common yarrow (9.1 %, Achillea millefolium).

Note §2: In treatments that received 75 kg N ha^-1^ yr^-1^ N was applied as mineral fertilizer split in two applications: 2/3 of total N in early spring (5 April) and 1/3 just after the 1^st^ biomass harvest (2 June). Grass300N had the fertilizer split in three: 2/5 in early spring and after the 1^st^ biomass harvest, and 1/5 after the 2^nd^ biomass harvest (5 August). All treatments received the same application of P (24 kg P ha^-1^ yr^-1^) and K (303 kg K ha^-1^ yr^-1^) divided in 2 applications (early spring and after 2^nd^ biomass harvest).

Table S2: Total nitrogen (N) and soil organic C (SOC) content in bulk soil and fractions in mg N g^-1^ dry soil and mg C g^-1^ dry soil. Fractions comprised “root fragments” (RF, >250 μm), particulate organic matter (POM, <250 μm >50 μm), and mineral-associated organic matter (MAOM, <50 μm). The % C recovery after fractionation was calculated as the sum of C in fractions relative to C in bulk soil. There were no statistical differences between treatments.

| **Treatment** | **N in  bulk soil  (mg N g^-1^ soil)** | **N in RF fraction  (mg N g^-1^ soil)** | **N in POM fraction  (mg N g^-1^ soil)** | **N in MAOM  fraction  (mg N g^-1^ soil)** | **SOC in  bulk soil  (mg C g^-1^ soil)** | **SOC in RF fraction  (mg C g^-1^ soil)** | **SOC in POM fraction (mg C g^-1^**  **soil)** | **SOC in MAOM fraction  (mg C g^-1^**  **soil)** | **% C  recovery** |
| --- | --- | --- | --- | --- | --- | --- | --- | --- | --- |
| Grass300N | 1.51 ± 0.07 | 0.20 ± 0.01 | 0.49 ± 0.01 | 6.17 ± 0.09 | 16.7 ± 0.8 | 0.71 ± 0.02 | 2.47 ± 0.10 | 12.68 ± 0.35 | 96 ± 3 |
| Grass75N | 1.49 ± 0.06 | 0.20 ± 0.02 | 0.49 ± 0.02 | 6.35 ± 0.04 | 16.4 ± 0.8 | 0.79 ± 0.10 | 2.37 ± 0.12 | 12.40 ± 0.40 | 95 ± 4 |
| Mix2 | 1.59 ± 0.04 | 0.22 ± 0.03 | 0.53 ± 0.05 | 6.34 ± 0.13 | 17.7 ± 0.6 | 0.86 ± 0.19 | 2.63 ± 0.30 | 12.83 ± 0.59 | 92 ± 1 |
| Mix6 | 1.58 ± 0.07 | 0.19 ± 0.01 | 0.51 ± 0.03 | 6.25 ± 0.09 | 17.4 ± 0.8 | 0.68 ± 0.04 | 2.50 ± 0.14 | 12.40 ± 0.25 | 90 ± 4 |
| Mix18 | 1.52 ± 0.09 | 0.18 ± 0.01 | 0.46 ± 0.02 | 6.30 ± 0.07 | 16.9 ± 1.1 | 0.66 ± 0.04 | 2.32 ± 0.12 | 12.81 ± 0.35 | 94 ± 3 |

Note: Mineral C was not detected in the soil, thus all soil C measured in bulk soil and fractions are considered soil organic C (WRB 2022; Yost and Hartemink, 2019).

- World Reference Base for Soil Resources. 2022. International Soil Classification System for Naming Soils and Creating Legends for Soil Maps, 4th edition. International Union of Soil Sciences (IUSS), Vienna, Austria.
- Yost J.L., Hartemink A.E. (2019) Soil organic carbon in sandy soils: A review. Advances in Agronomy. pp. 217-310. DOI: 10.1016/bs.agron.2019.07.004.

Tabel S3: The proportions of each functional group (grass, legume, forb) of the total aboveground biomass harvest in 2022. The percentage of N derived from the atmosphere was measured (%Ndfa) in legume biomass. Mean values derived from the measured data, ± indicates standard error (n = 4).

| **Treatment** | **Aboveground biomass Yield (t DM ha^-1^)** | **Legume %** | **Grass %** | **Forb %** | **Unsown species %** | **%Ndfa** | |
| --- | --- | --- | --- | --- | --- | --- | --- |
| *Grass300N* | *18.1 ± 0.7* | - | *99 ± 0* | - | *1 ± 0* | *-* |  |
| Grass75N | 6.8 ± 0.5 (a) | - | 95 ± 1 (c) | - | 5 ± 1 (b) | - |  |
| Mix2 | 14.3 ± 0.6 (b) | 63 ± 4 (a) | 34 ± 4 (b) | - | 3 ± 1 (ab) | 98 ± 1 % |  |
| Mix6 | 19.7 ± 1.0 (c) | 59 ± 4 (a) | 18 ± 3 (a) | 21 ± 4 (b) | 2 ± 1 (a) | 99 ± 0.2 % |  |
| Mix18 | 19.2 ± 1.3 (c) | 56 ± 6 (a) | 31 ± 7 (b) | 11 ± 2 (a) | 2 ± 1 (a) | 97 ± 1 % |  |

Note: The ^15^N isotope dilution method was used to quantify the biological N_2_ fixation by the legume species, using the non-legume component as a reference crop. Prior to the growing period (2 March), each labelling plot received ^15^N-labelled ammonium sulphate ((NH₄)₂SO₄) (98 atom %) corresponding to 1 kg N ha^-1^. Thereby, the proportion of N derived from the atmosphere (%Ndfa) was calculated as the difference in ^15^N enrichment between the legume and the non-legume, where the atom% ^15^N excess is the difference between ^15^N enrichment of legume or reference crop in the labelled plots and the natural abundance of ^15^N in the non-labelled legume or reference crop in adjacent subplots.

$\%Ndfa=\left( 1-\frac{atom\% {}^{15}N excess in legume crop}{atom\% {}^{15}N excess in reference crop} \right)*100$

Table S4: Total root biomass to 25 cm depth, root fragments, and the content of nutrient detergent fibers in root biomass DM (lignin, cellulose and hemicellulose). Mean values derived from the measured data, ± indicates standard error (n = 4). Compact letter display indicates differences between treatments derived from post hoc comparisons.

| **Treatment** | **Lignin %** | **Cellulose %** | **Hemicellulose %** | **Total NDF %  of root DM** | **Root biomass 0-25 cm g DM kg^-1^ dry soil** | **Root fragments 0-25 cm**  **g DM kg^-1^ dry soil** |
| --- | --- | --- | --- | --- | --- | --- |
| *Grass300N* | *5.2 ± 0.1* | *26.7 ± 0.7* | *29.0 ± 0.6* | *60.9 ± 1.3* | *2.25 ± 0.10* | *0.21 ± 0.03* |
| Grass75N | 4.7 ± 0.2 (a) | 24.3 ± 1.0 (ab) | 22.7 ± 2.0 (b) | 51.8 ± 2.8 (ab) | 1.61 ± 0.16 (b) | 0.23 ± 0.03 (b) |
| Mix2 | 6.2 ± 0.1 (b) | 26.8 ± 1.1 (b) | 20.9 ± 1.2 (b) | 53.9 ± 1.1 (b) | 1.20 ± 0.11 (a) | 0.17 ± 0.04 (ab) |
| Mix6 | 6.4 ± 0.6 (b) | 21.9 ± 1.2 (a) | 14.6 ± 0.6 (a) | 42.9 ± 1.4 (a) | 2.19 ± 0.16 (c) | 0.17 ± 0.00 (ab) |
| Mix18 | 6.2 ± 0.6 (b) | 25.0 ± 1.8 (ab) | 16.9 ± 2.6 (ab) | 48.0 ± 4.8 (ab) | 1.33 ± 0.15 (a) | 0.11 ± 0.01 (a) |

Table S5: Proportion of mineral-associated organic carbon (%MAOC) and particulate organic carbon (%POC), as well as the quantity of each fraction (qMAOC and qPOC) from the total quantity of C lost via rhizodeposition from the living plant to the soil (qClvR). Mean values derived from the measured data, ± indicates standard error (n = 4). Compact letter display indicates differences between treatments derived from post hoc comparisons.

| **Treatment** |  | **%MAOC** | **%POC** | **qMAOC g C kg^-1^ dry soil** | **qPOC g C kg^-1^ dry soil** | **qClvR g C kg^-1^ dry soil** |
| --- | --- | --- | --- | --- | --- | --- |
| *Grass300N* |  | *66 ± 3* | *34 ± 3* | *0.15 ± 0.03* | *0.08 ± 0.02* | *0.23 ± 0.05* |
| Grass75N |  | 62 ± 2 (a) | 38 ± 2 (b) | 0.21 ± 0.01 (b) | 0.13 ± 0.01 (b) | 0.33 ± 0.02 (b) |
| Mix2 |  | 69 ± 2 (b) | 31 ± 2 (b) | 0.12 ± 0.01 (a) | 0.06 ± 0 (a) | 0.18 ± 0.01 (a) |
| Mix6 |  | 68 ± 1 (b) | 32 ± 1 (a) | 0.10 ± 0.02 (a) | 0.05 ± 0.01 (a) | 0.15 ± 0.02 (a) |
| Mix18 |  | 66 ± 1 (ab) | 34 ± 1 (ab) | 0.12 ± 0.02 (a) | 0.06 ± 0.01 (a) | 0.18 ± 0.04 (a) |


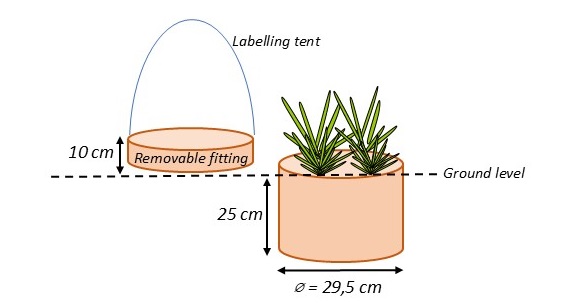
*Figure S1: Isotopic pulse-labelling with ^13^C-CO2 was performing during the entire growing season in PVC-cylinders inserted 25 cm into the soil with an inner diameter of 29.5 cm. A PVC-fitting was used during the season for attachment of the labelling tent, but the fitting was removed during harvest.*

*
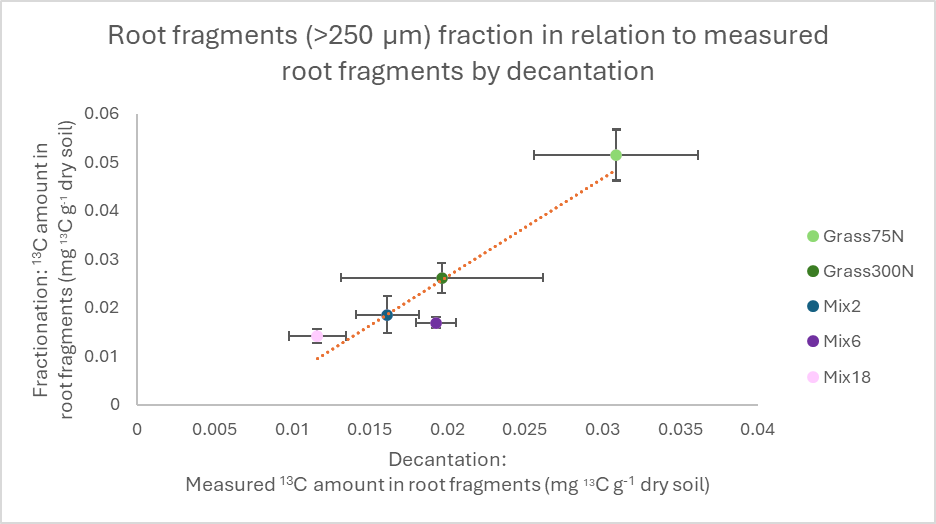
*

*Figure S2: The SOC fraction >250 µm was defined as root fragments in the present study, and this fraction correlate (p-value = 0.046; R = 0.45) with the root fragments determined in the same treatments by wet sieving using similar mesh size (>250 µm) and decantation.*


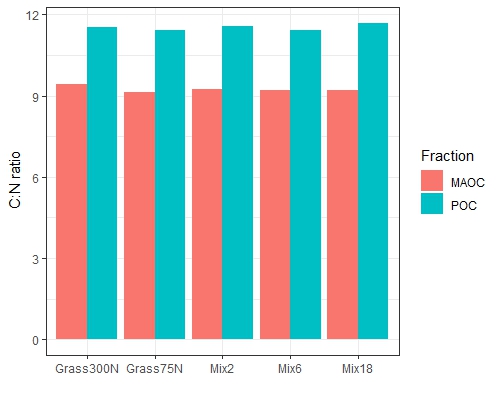


*Figure S3: The C:N ratios of the SOC fractions show qualitative differences between the fractions. These C:N ratios are based on the total C and N in SOC fractions – not only the recently formed C. Across all treatments C:N ratio of the MAOC fractions (9.25 ± 0.11) was significantly lower (p<0.001) than C:N ratio of the POC fractions (mean 11.53 ± 0.13).*


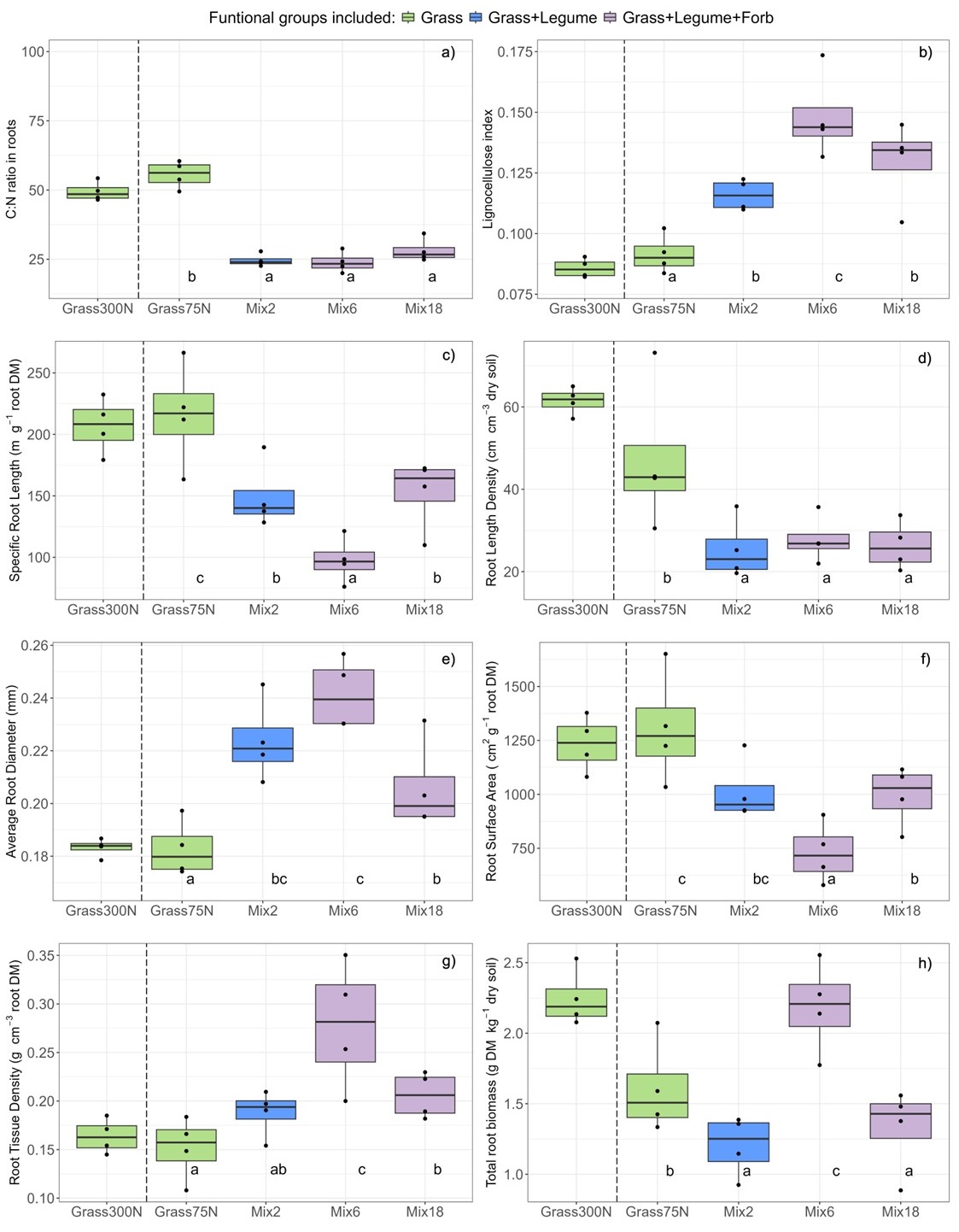


*Figure S4: Treatment differences in morphological and biochemical root traits, and total root biomass. Treatments: Grass300N = Perennial ryegrass with 300 kg N ha^-1^ yr^-1^, Grass75N = Perennial ryegrass with 75 kg N ha^-1^ yr^-1^, Mix2 = perennial ryegrass and white clover, Mix6 and Mix18 = Mixtures with 6 and 18 species, see table S1 for all species. Error bars indicate standard error (n = 4). Compact letter display indicates differences between mixtures with the same low fertilizer rate (75 kg N ha^-1^ yr^-1^) derived from post hoc comparisons. Functional groups: Gr=grass, Le=Legume, Fo=Forb. If including all treatments regardless of fertilization in the model, the following pairwise comparisons were observed:*

*Specific root length (SRL) was significantly lower for Mix6 than for Grass75N (p<0.001) and Grass300N (p=0.001). The SRL in Mix2 was only lower than Grass75N (p=0.047) with a strong tendency to be lower than in Grass300N (p=0.097), and Mix18 showed tendencies to be lower than both Grass75N (p=0.061) and Grass300N (p=0.130).*

*Root length density (RLD) was significantly higher for Grass300N than all mixtures (Mix2 (p<0.001), Mix6 (p=0.002) and Mix18 (p=0.001), and Grass75 had a significantly higher RLD than Mix2 (p=0.032) and Mix18 (p=0.042), with a strong tendency for higher RLD than the Mix6 (p=0.062).*

*Average root diameter (ARD) was significantly higher for Mix6 compared to both Grass75N (p<0.001) and Grass300N (p<0.001), and for Mix2 compared to Grass75 (p=0.002) and Grass300N (p=0.003), but there was only a strong tendency observed for Mix18 when compared to Grass75N (p=0.087) and Grass300N (p=0.096).*

*Root surface area (RSA) was significantly higher in Grass300N compared to Mix6 but not different compared to Mix2 and Mix18. RSA was similar between Grass300N and Grass75N.*

*The root tissue density (RTD) in Mix6 was significantly higher than Grass75N, Grass300N and Mix2, and tended to be higher than in Mix18 (p=0.095), while the RTD was similar between the other treatments.*


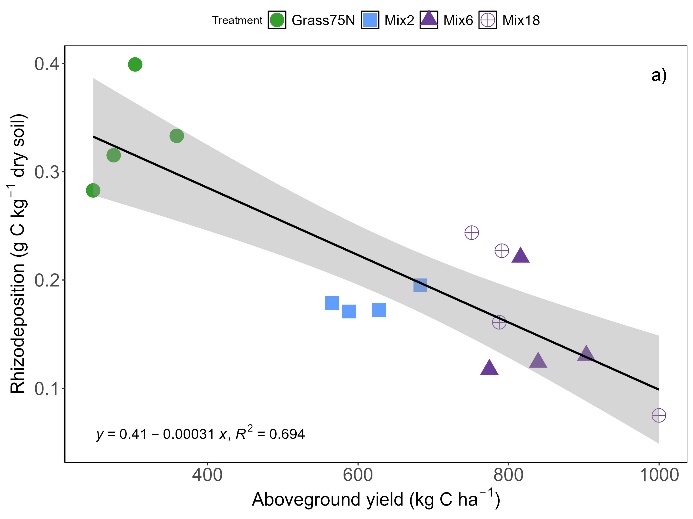

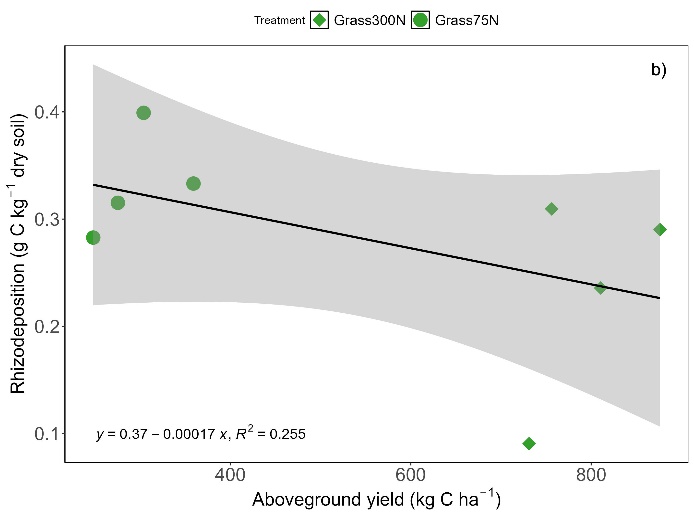


*Figure S5: Linear regression models showing the relationship between aboveground biomass yield (kg C ha^-1^) and net rhizodeposition (g C kg dry soil^-1^) across (a) treatments at low N fertilizer application rate (χ^2^_1_=28.14, p<0.001, and (b) treatments of monoculture perennial ryegrass with high and low N fertilization application rate (χ^2^_1_=2.69, p<0. 0.101).*


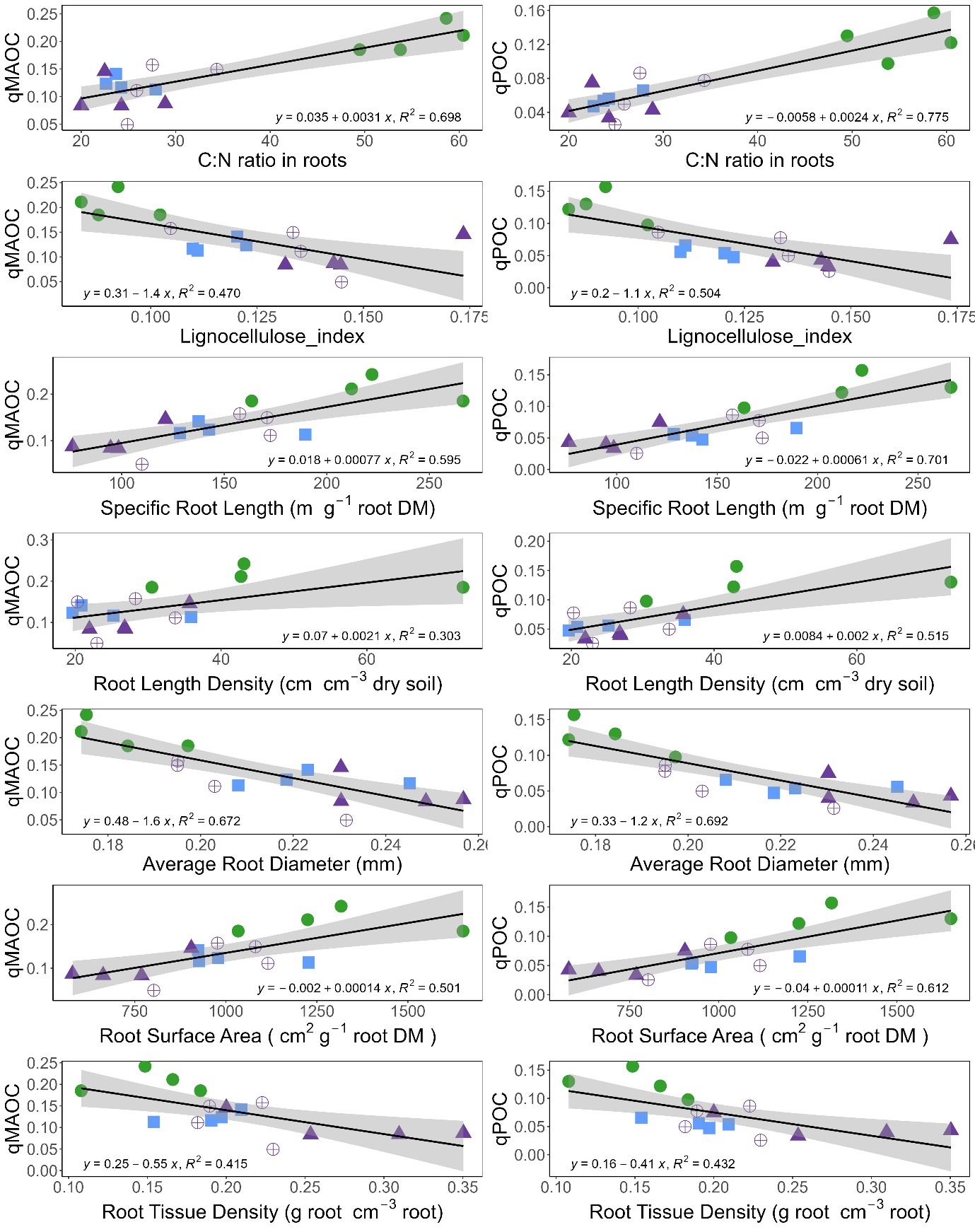
*Figure S6: Linear regressions with individual root traits and quantity of MAOC vs POC (qMAOC and qPOC respectively). The two fractions were both well represented by the overall regressions with total C rhizodeposition (Figure 3) and root traits. Treatments: Grass75N (green circles) = Perennial ryegrass with 75 kg N ha^-1^ yr^-1^, Mix2 (blue squares) = perennial ryegrass and white clover, Mix6 (purple triangles) and Mix18 (purple crossed circles) = Mixtures with 6 and 18 species, see table S1 for all species. All mixtures fertilized with 75 kg N ha^-1^ yr^-1^.*


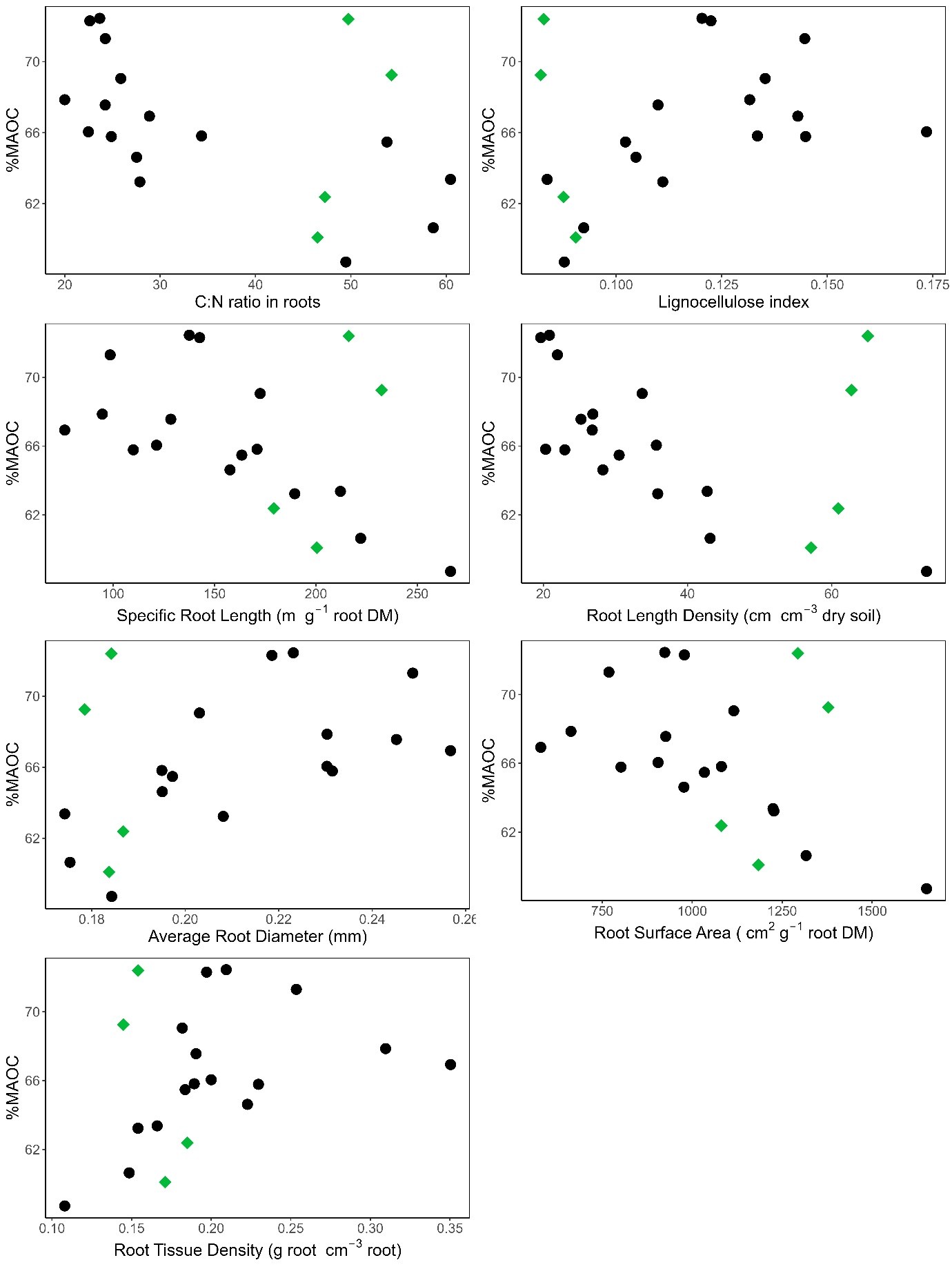
*Figure S7: Linear regressions between individual root traits and %MAOC were hampered or weakened when including the reference treatments Grass300N in the model, compared to when regression models only contained low-fertilized treatments (Figure 4). Green diamonds = Grass300N, black circles = treatments with 75 kg N fertilizer. Including Grass300N in linear regressions with %MAOC and the individual root traits: C:N ratio in root biomass (F_1,18_=5.20, p=0.035, R^2^=0.22), lignocellulose index (F_1,18_= 2.47, p=0.136, R^2^=0.11), SRL (F_1,18_=4.84, p=0.041, R^2^=0.20), RLD (F_1,18_=4.24, p=0.054, R^2^=0.18), ARD (F_1,18_=4.83, p=0.041, R^2^=0.20), RSA (F_1,18_=4.27, p=0.053, R^2^=0.18), and RTD: (F_1,18_=2.36, p=0.142, R^2^=0.11).*


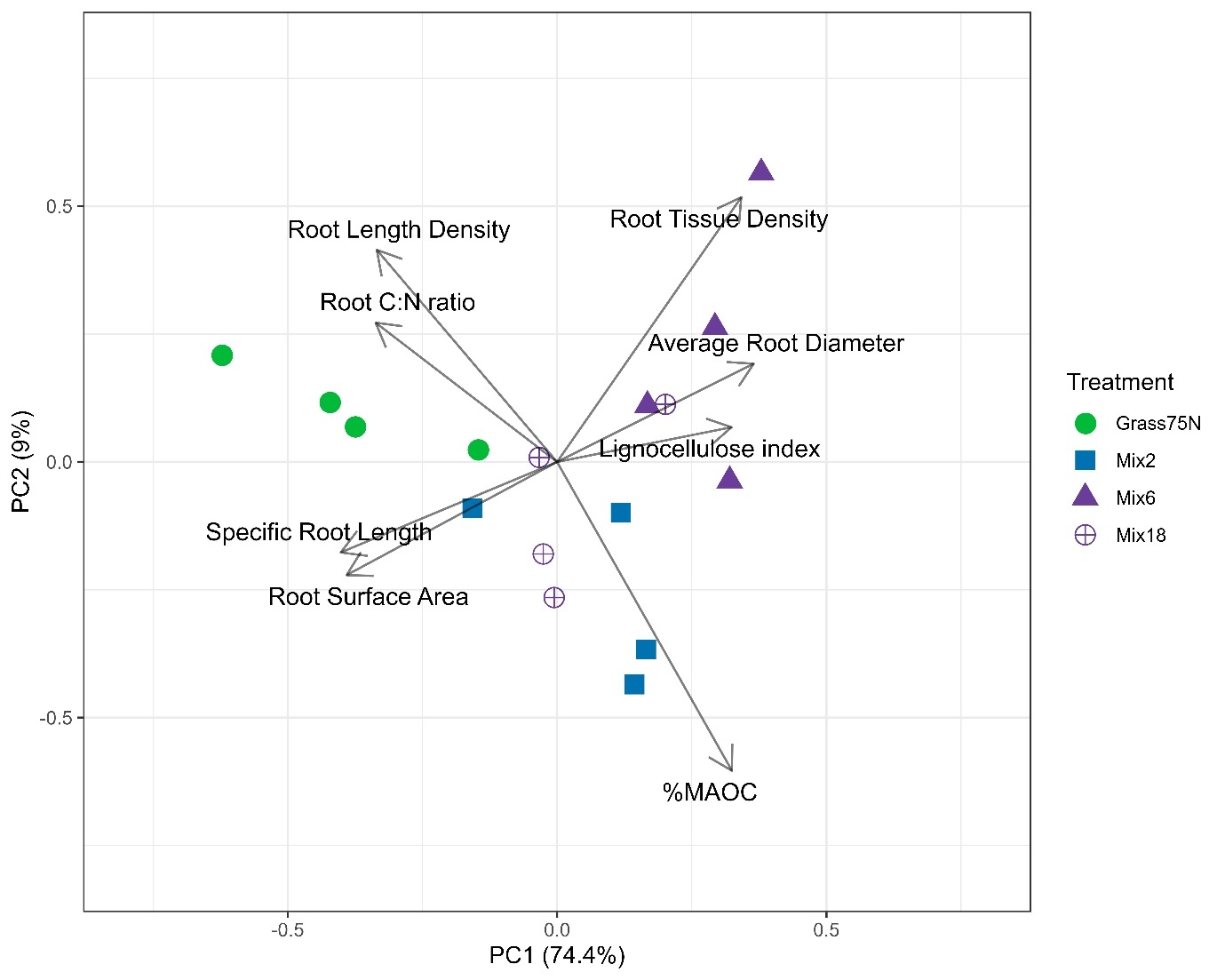


*Figure S8: Princ**ipal Component Analysis (PCA) with all root traits and the relative proportion of mineral-associated organic carbon (%MAOC), with the proportion of Particulate Organic Carbon (POC) as the inverse of %MAOC as the two add up to 100%. The treatments as indicated by symbols in the PCA are: Grass75N = Perennial ryegrass with 75 kg N ha^-1^ yr^-1^, Mix2 = Perennial ryegrass and white clover, and Mix6 and Mix18 = mixtures with 6 and 18 species, see table S1 for all species.*
